# Artificial intelligence in the radiologic assessment of ductal carcinoma *in situ*: a systematic review

**DOI:** 10.3389/fonc.2026.1870768

**Published:** 2026-07-14

**Authors:** Colin Wu, Alison Bartak, Ramaswamy Sharma

**Affiliations:** 1 University of the Incarnate Word School of Osteopathic Medicine, San Antonio, TX, United States; 2 Baptist University College of Osteopathic Medicine, Memphis, TN, United States

**Keywords:** artificial intelligence, convolutional neural network, DCIS, deep learning, ductal carcinoma *in situ*, machine learning

## Abstract

Ductal carcinoma *in situ* (DCIS) accounts for approximately 25% of new breast cancer diagnoses and carries a 20% to 50% risk of post-surgical upstaging to invasive cancer, complicating risk stratification and raising concerns about overtreatment. Mammography, the primary imaging modality, is limited by low sensitivity in dense breasts and low-grade lesions. Supplemental imaging (ultrasound, magnetic resonance imaging) can improve detection but is often costly and less accessible. Artificial intelligence (AI) has emerged as a promising approach to enhance DCIS radiologic assessment. A systematic search of several databases was conducted to determine the role of AI technologies in supporting radiological assessment of DCIS following PRISMA guidelines. Of 311 studies identified, 46 met inclusion criteria. AI shows substantial promise in improving detection, classification, preoperative risk stratification, and molecular inference, with area under the curves (AUCs) ranging from 0.70 to 0.97, sensitivities of 80–96%, and specificities up to 93%. Overall, temporal, multiphase, and spatially aware models outperformed conventional 2D approaches. The findings underscore the potential for clinical integration, inform best practices, and identify critical gaps to guide future studies and development of standardized, validated AI tools for DCIS management. Limitations included retrospective design, small cohort of DCIS-specific datasets, minimal external validation, generalizability concerns, and weaker performance relative to invasive breast cancer.

## 1 Introduction

Ductal carcinoma *in situ* (DCIS) is an early-stage malignancy characterized by the proliferation of cancerous epithelial cells confined within mammary ducts, without invasion into stromal tissue. Although considered stage 0 breast cancer, DCIS carries a significant yet non-obligate 20% to 50% risk of being upstaged to invasive breast cancer at surgery (1), reflecting occult invasion present at biopsy but missed due to sampling limitations, thereby necessitating accurate preoperative risk stratification. Importantly, recent studies show that only a subset of DCIS cases progress to invasive breast cancer, a distinct biological transition that occurs over time, and surgical treatment does not significantly improve survival (1, 2). Yet, diagnostic uncertainty often leads to overtreatment, with standard management typically resembling those for invasive breast cancer, including surgical excision followed by adjuvant radiation or endocrine therapy to avoid missing an occult invasive component (2). Thus, the key challenge to management of DCIS is accurate diagnosis and identification of patients who truly require aggressive treatment from those with low-risk disease for whom intervention may be unnecessary.

The use of imaging, primarily mammography, is fundamental for the detection of DCIS. With the introduction of standardized breast screening programs and increased access to mammography screening, the estimated number of new DCIS cases in the United States has steadily increased over time, from 56,500 in 2024 to 59,080 in 2025 and 60,730 in 2026 (3). However, the accuracy of mammography is limited by factors such as dense breast tissue, and the subtle appearance of lower-grade lesions, prompting the need for ultrasound or magnetic resonance imaging (MRI) that may be prohibitive in terms of cost and/or access (4–7).

Artificial intelligence (AI) has emerged as a promising, rapidly advancing tool capable of enhancing diagnostic accuracy, sensitivity, and specificity (8–10). AI models employing traditional machine learning algorithms as well as newer, deep learning (DL) convolutional neural networks (CNN) have been evaluated in applications that automate and enhance DCIS detection, discriminate between invasive ductal carcinoma (IDC), atypical ductal hyperplasia (ADH) and DCIS, predict upstaging risk, and provide real-time intraoperative margin assessments. Thus, AI has the potential to improve nearly every aspect of DCIS diagnosis and management (7, 11–15).

Although prior reviews have addressed AI applications in breast imaging more broadly, the evidence base specific to DCIS remains distinct, as this condition presents unique challenges such as subtle imaging findings, occult invasion, upstaging risk, overtreatment, and limited availability of dedicated datasets. Existing reviews (7) provide important context for AI in breast cancer imaging, but they do not comprehensively synthesize the heterogeneous DCIS-focused literature across detection, lesion classification, upstaging prediction, molecular inference, margin assessment, recurrence prediction, and clinical translation. Accordingly, this systematic review aims to comprehensively evaluate current evidence on AI’s application in relation to DCIS imaging, with a focus on diagnostic performance, clinical implications, and existing limitations.

## 2 Methods

### 2.1 Search strategy

This systematic review was conducted according to PRISMA 2020 guidelines. On February 13, 2025, three databases, MEDLINE Ovid, PubMed, and Web of Science, were queried for relevant publications using the search string: (“artificial intelligence” OR AI OR “machine learning” OR “deep learning” OR “natural language processing”) AND (“radiology” OR “radiography” OR “mammography” OR “CT” OR “computed tomography” OR “X ray” OR “X-ray” OR “xray” OR “MRI” OR “magnetic resonance imaging” OR “ultrasound” OR “sonography” OR “US”) AND (“DCIS” OR “ductal carcinoma in situ”). MEDLINE Ovid, PubMed, and Web of Science were selected to capture the biomedical, radiology, oncology, and multidisciplinary literature most directly relevant to AI-based radiologic assessment of DCIS, consistent with database approaches used in prior AI radiology reviews. No publication date restriction was applied.

The search was repeated on June 12, 2025, using an expanded search strategy that added ultrasound-related terms: (“artificial intelligence” OR AI OR “machine learning” OR “deep learning” OR “natural language processing”) AND (“radiology” OR “radiography” OR “mammography” OR “CT” OR “computed tomography” OR “X ray” OR “X-ray” OR “xray” OR “MRI” OR “magnetic resonance imaging” OR “ultrasound” OR “sonography” OR “US”) AND (“DCIS” OR “ductal carcinoma in situ”). All references retrieved were imported into Zotero for reference management, duplicate removal, and screening.

### 2.2 Inclusion and exclusion criteria

Studies were included if they incorporated at least one AI-related methodology, including machine learning, deep learning, radiomics, or computer-aided image analysis, that was applied to a radiologic imaging-based modality such as mammography, ultrasound, magnetic resource imaging (MRI), computed tomography (CT), optical coherence tomography (OCT), or photoacoustic imaging, and addressed at least one of the following clinical aspects of DCIS: detection or diagnosis, risk stratification, discrimination from closely resembling neoplasia such as ADH or DCIS with microinvasion, prediction of upstaging or recurrence after DCIS resection, prognostic assessment, treatment planning, intraoperative margin assessment, or survival. Multimodal studies incorporating clinical, pathologic, or histopathologic variables were eligible only when a radiologic or image-based modality was a central input and the study addressed a DCIS-relevant diagnostic, prognostic, or management question. Eligible study designs included retrospective, prospective, or observational original research articles involving human subjects and published in peer-reviewed journals.

Because the available literature varied substantially in its degree of DCIS specificity, included studies were stratified during data extraction into two evidence categories. Studies were considered direct DCIS-specific evidence if they included a DCIS cohort and reported DCIS-specific outcomes, endpoints, or performance metrics. Studies were considered contextual evidence if they evaluated broader breast lesion or breast cancer imaging tasks that included DCIS within the study population, compared DCIS with invasive breast cancer or other breast neoplasia, addressed an imaging problem directly relevant to DCIS management, or provided supportive information for interpreting DCIS-related AI performance in relation to adjacent breast cancer tasks. Contextual studies were included to allow comparison of AI performance across DCIS, invasive breast cancer, and related breast lesion classification tasks, but were interpreted separately from direct DCIS-specific evidence and were not treated as substitutes for DCIS-specific validation.

Studies were excluded if they did not address DCIS or DCIS-relevant imaging or management question or did not apply AI-based methods to radiologic imaging-based assessment, or lacked an imaging or image-derived input. Non-peer-reviewed articles, publications that did not present original research data such as reviews, editorials, or commentaries, conference abstracts, single-patient case studies, and studies that were based on non-human or pre-clinical studies were also excluded. Finally, studies that were not published in English or their full-text articles could not be accessed were excluded.

### 2.3 Study selection

After removing duplicate results, two reviewers independently screened study titles and abstracts for relevance based on the predefined inclusion and exclusion criteria. Non-relevant articles were removed, and the full text of the remaining articles was retrieved; articles that could not be accessed through the institutional library, interlibrary loan, or by direct communication with the corresponding author were excluded. Retrieved full-text articles were reviewed again for eligibility. Any disagreements during title/abstract screening or full-text eligibility assessment were resolved through discussion between two reviewers, with a third reviewer available for adjudication if consensus could not be reached. Data were extracted using a standardized form that included study design, cohort size, DCIS sample size, imaging modality, AI method, clinical task, validation strategy, performance metrics, and key limitations. Because of substantial heterogeneity in clinical tasks, imaging modalities, AI methods, endpoints, validation strategies, and reported performance metrics, findings were synthesized narratively rather than by meta-analysis.

### 2.4 Risk of bias and quality assessment

The methodological quality and risk of bias of included studies were independently assessed using QUADAS-2 (Quality Assessment of Diagnostic Accuracy Studies-2) (16), which was selected because most included studies reported diagnostic accuracy-type outcomes. Although QUADAS-2 does not fully capture AI-specific issues such as data leakage, overfitting, calibration, and external validation, these concerns were considered qualitatively when interpreting the findings. The results of the assessment (**Supplementary Table 1**) were used to inform the interpretation of findings.

## 3 Results

### 3.1 Search results

**Figure 1:**
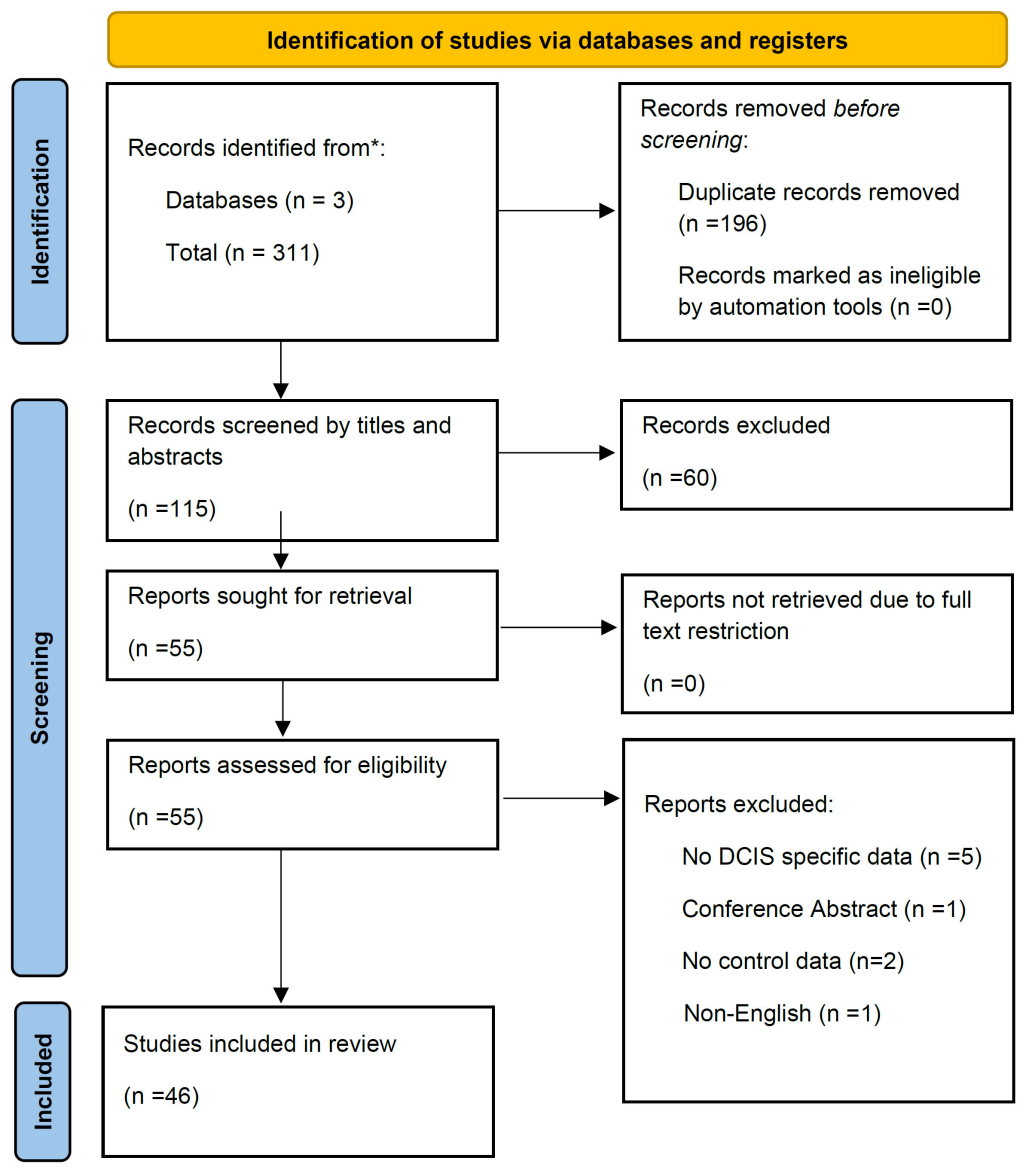
PRISMA 2020 diagram illustrating the study selection process of this systematic review.

A total of 311 studies were identified across the three databases using the search terms (**Figure 1**). After removing 196 duplicate entries, 115 unique records remained. Screening of titles and abstracts identified 55 articles as meeting the inclusion criteria and their full-text copies were retrieved. Of these, nine articles were excluded: two articles that lacked control data, one non-English publication, one conference abstract, and five articles that were not DCIS-specific. Therefore, 46 full-text articles were included in this systematic review. The characteristics of included studies are summarized in **Table 1**. Most studies demonstrated high risk of bias in patient selection due to retrospective or non-representative sampling strategies, while other domains were largely low risk, and applicability concerns were minimal.

**Table 1:** Key characteristics and findings of studies included in this review.

Author, Year	Cohort (n)	Imaging modality	AI model	Performance
Detection				
Raafat et al., 2022 (17)	123 patients (9 DCIS)	Mammography	AI-CAD	Compared AI to mammography for cancer detection; AI sensitivity: 96.6% overall and 100% for DCIS; mammography sensitivity: 87.3% overall and 88.9% for DCIS
Waugh et al., 2024 (18)	7,533 patients (13 DCIS)	Mammography	DL-CNN	Compared AI to radiologists: AI sensitivity: 94% overall and 92.3% for DCIS; Radiologists: 100%; overall and 100% DCIS
Weigel et al., 2023 (19)	634 patients (644 lesions; 151 DCIS, 44 with invasive cancer)	Mammography	DL-CNN	Assessed calcification malignancy risk on mammography; overall AI false-negative rate was 7.2%. False-negative rate higher for DCIS (8.6%; 13/151) compared with invasive cancer (2.3%; 1/44); did not discriminate well between DCIS and benign lesions
Lee S. E. et al., 2022 (20)	896 patients (930 cancerous breasts; 192 DCIS)	Mammography	AI-CAD	Evaluated AI-CAD abnormality scores in breast cancer depiction; AI false-negative rate was 19.4% (180/930) overall and 28.9% (52/192) DCIS
Xiao et al., 2019 (21)	448 lesions (29 DCIS)	Ultrasound	DL-CAD	Distinguished benign from malignant breast lesions; correctly classified 88.1% of fibroadenomas (119/135) and 71.4% of adenosis (25/35). However, AI diagnostic accuracy for DCIS was 72.4% (21/29) compared to 96.6% (28/29) for the experienced radiologist and 86.2% (25/29) for the resident.
Ma et al., 2024 (22)	196 patients (202 lesions; 10 DCIS)	Ultrasound	DL-CNN	Detected and triaged incidental breast masses on ultrasound; AI improved diagnostic sensitivity from 70% to 80% for radiologists with 23, 10, and 3 years of experience but this was not statistically significant.
Lee K. E. et al., 2025 (60)	501 patients (62 DCIS)	Mammography	AI-CAD	Compared AI-CAD performance on synthetic mammography (AUC = 0.86) and FFDM (AUC = 0.87); DCIS sensitivity was 61.3% vs 77.4% respectively.
Condon et al., 2024 (57)	959 patients (85 DCIS, 425 malignant, 44 benign, 490 controls)	Mammography	DL-CNN	AUC decreased from 0.83 to 0.76 when models were trained and tested on different datasets (NYU and Australian datasets, respectively); AUCs improved with retraining on local dataset.
Berg et al., 2023 (59)	300 patients (758 lesions; 6 DCIS)	Ultrasound	AI software on portable and standard US images	Triage of palpable breast masses using portable and standard ultrasound images; specificity was lower with portable (48%) compared with standard (79%) ultrasound. The AI software achieved AUC of 0.91–0.95 and sensitivity 96%–98% for cancer detection. Portable US vs SOC US: AUC of 0.91 vs 0.95; specificity 48% vs 79%; performance dropped with minimally trained observers (AUC of 0.78)
Hsu et al., 2022 (58)	37,317 exams (114 DCIS)	Mammography	Ensemble DL model	Externally validated an ensemble DL model composed of 11 top-performing AI models for automated mammography interpretation; it underperformed compared with radiologist assessment, but performance improved when used alongside radiologist interpretation. Ensemble model vs radiologists (P < 0.001): Sensitivity, 0.547 vs. 0.826; Specificity: 0.697 vs. 0.930
Discrimination (DCIS vs. IDC)				
Yin H. et al., 2021 (27)	802 patients (347 DCIS)	MRI	CNN	Distinguished DCIS from IDC using apparent diffusion coefficient (ADC)-based MRI; CNNs trained on ADC maps significantly outperformed traditional mean ADC measurements. Internal test set, AUC 0.977 vs 0.866; external test set, AUC 0.926 vs 0.845; reduced prediction time from approximately 3–8 min for manual ADC to one second per lesion
Jayender et al., 2013 (25)	41 cases (22 IDC, 19 DCIS)	MRI	Statistical Learning Algorithm for Tumor Segmentation (SLATS) combining hidden Markov models, Fast Fourier Transform, Short-Time Fourier Transform, and fuzzy c-means clustering	Compared statistical learning approaches for tumor segmentation on DCE-MRI; DCIS vs IDC accuracy: 79.3% vs. 92.6% with 100% sensitivity for both
Nassif et al., 2012 (26)	1,587 mammograms (222 DCIS; stratified by age)	Mammography	Logical Differential Prediction-Bayes Net (LDP-BN).	Improved diagnostic accuracy and reduced overdiagnosis in older women using mammography; AUCs of age-specific and standard traditional Bayes Net model were 0.8911 and 0.8304 respectively,
Zhang et al., 2024 (28)	629 patients (train 363, validation 91, test 175)	Ultrasound	Radiomics	Distinguished IDC from DCIS using ultrasound radiomics; the model achieved an external test AUC of 0.770.
Discrimination (DCIS vs. Benign Tumors)				
Ha et al., 2019 (30)	149 patients (67 ADH, 82 DCIS)	Mammography	CNN	Distinguished ADH from DCIS on mammography; the CNN achieved an AUC of 0.86, accuracy of 86.7%, sensitivity 84.6%, and specificity of 88.2%
Mutasa, Chang, Nemer, et al., 2020 (15)	140 patients (61 ADH, 79 DCIS)	Mammography	CNN	Distinguished ADH from DCIS; strong specificity in reducing DCIS–ADH misclassification. the CNN achieved an AUC of 0.90, accuracy of 80.7%, sensitivity of 63.9%, and specificity of 93.7%.
Yin J. et al., 2023 (31)	1,112 patients (461 DCIS, 651 fibroadenoma; test: 139 DCIS, 181 fibroadenoma)	Ultrasound	DL + ML	Developed models effective in differentiating DCIS from fibroadenoma; trained ML (SVM model), DL, and a difference-based self-supervised (DSS) model; achieved AUCs of 0.794 (SVM), 0.766 (DL), and 0.817 (DSS)
Jiang et al., 2024 (32)	306 patients (198 DCIS, 108 fibromatoses; training 184, validation 122)	Ultrasound + clinical data	ML	Distinguished DCIS from breast fibromatosis using clinical radiomics; a combined clinical radiomics model achieved an AUC of 0.983–0.996, outperforming individual radiomics and clinical ultrasound models
Grade Classification				
Wu Y. et al., 2025 (33)	237 patients (241 DCIS)	Multimodal imaging + clinical data	Ensemble ML model	Predicted low nuclear grade DCIS using ensemble ML model integrating clinical data, ultrasound images, mammography images, and radiomic scores; AI outperformed clinical data only model with AUCs of 0.92 vs. 0.86 respectively, improving discrimination and reclassification.
Alaeikhanehshir et al., 2024 (5)**	464 patients (854 mammograms; DCIS only)	Mammography	U-Net CNN	Discriminated low- from high-grade DCIS and higher-risk disease for active surveillance exclusion; AUC of 0.72 when classifying DCIS as high risk; improved to 0.76 when discriminating between upstaged/high risk DCIS from low-risk DCIS
Wu H. et al., 2024 (1)**	733 patients (754 DCIS; N1 = 141, N2 = 33)	Multimodal imaging + clinical/pathologic data	DL + ML	Classified DCIS grade and upstaging status using multimodal data. Multimodal model accuracy and AUC of discriminating low-grade DCIS from upstaged low-grade DCIS were 0.769–0.987 and 0.818–0.939, respectively; accuracy and AUC for discriminating DCIS from upstaged DCIS were 0.751–0.780 and 0.829–0.86, respectively. Thus, the model can discriminate DCIS from upstaged DCIS as well as between low-grade DCIS and upstaged low-grade DCIS.
Liu Y. et al., 2024 (34)	1,760 patients (61 DCIS)	MRI	ML-based unenhanced radiomics model	Classified breast cancer on unenhanced MRI using radiomics; the model had a diagnostic accuracy similar to that of a combined radiomics model based on T2W, DW, and first contrast-enhanced T1-weighted sequence, as well as radiologists and provided full multiparametric protocol MRI results. The model achieved AUCs of 0.893 (training), 0.848 (validation), and 0.677 (sensitivity).
Clinical Decision Support				
Barros et al., 2023 (35)	9,234 patients (multi-cohort with 3017 Israeli women and 2336 US women; includes 3,881 pretraining cases)	Mammography + EHR	CNN + supervised learning	Identified malignant breast lesion subtypes. Model validated and tested using Israeli and US cohorts; overall AUC of 0.80 (US), 0.88 (Israel); DCIS-specific AUC of 0.74 (US), 0.76 (Israel). At 99% sensitivity, the model reduced unnecessary biopsies by 13% while missing only 1.3% of malignancies.
Liu C. et al., 2024 (36)	847 patients (200 DCIS)	Mammography	CNN	Reduced unnecessary biopsy in BI-RADS 4 lesions using mammographic CNN models. Discriminated between benign vs DCIS with AUC of 0.73 and specificity of 25% at 95% sensitivity. Model show comparable performance distinguishing benign from invasive lesions and improved performance in distinguishing benign lesions from atypia (AUC 0.70) and benign lesions from invasive tumors (AUC 0.72).
Risk Assessment				
Lee S.E., 2024 (6)	126 patients (30 DCIS)	Mammography	AI-CAD	Tracked AI-CAD abnormality scores on serial mammography before cancer diagnosis; AI abnormality score showed increased trend for invasive cancers (+1.4 per 6 months, p=0.002) that was not observed for DCIS (p=0.771) or occult cancers. Thus, AI-CAD showed limited sensitivity for DCIS and occult malignancies.
Damiani et al., 2023 (41)	2,740 patients (557 DCIS)	Mammography	DL	Estimated 3-year breast cancer risk after a negative screening mammogram; the model achieved an AUC of 0.68 for 3-year risk prediction of breast cancer. No significant difference in prediction between invasive cancer vs DCIS, with AUC of 0.68 and 0.66 respectively.
Santeramo et al., 2024 (42)	3,386 matched pairs (420 DCIS)	Mammography	DL	Compared AI models for cancer risk and detection using paired mammograms at diagnosis and previous screening visit; risk AUC ranged from 0.59 to 0.67 and detection AUC from 0.81 to 0.89.
Manley et al., 2021 (43)	541 patients (206 DCIS)	Mammography	CNN	Assessed whether CNN risk scores reflected chemoprevention response in high-risk lesions; women receiving chemoprevention showed decreased risk scores (33.7% to 22.9%). Thus, model is useful for personalized risk and treatment monitoring.
Upstaging, Microinvasion, and Invasive Disease Classification				
Mutasa, Chang, Van Sant, et al., 2020 (44)	123 patients (82 DCIS, 41 invasive)	Mammography	CNN	Predicted occult invasive upgrade in DCIS from mammographic radiomics; the model achieved an AUC of 0.71, accuracy of 74.6%, and specificity of 91.6% for discriminating patients with DCIS from DCIS with invasion
Hou et al., 2022 (12)	700 patients (114 upstaged DCIS)	Mammography	Radiomics + logistic regression	Best model combining radiomic and clinical features achieved AUC 0.71; sensitivity 37% at fixed 90% specificity; similar performance with combined radiomic and clinical features with selection (AUC 0.68) or the radiomics-only model with selection (AUC 0.69). Radiomics with clinical features outperformed clinical alone (p < 0.05).
Shi et al., 2018 (45)	99 patients (25 upstaged DCIS)	Mammography	Deep features + SVM	Predicted pre-surgical DCIS upstaging from mammographic deep features; the CNN achieved an AUC of 0.70 vs. handcrafted model with AUC of 0.68.
Hou et al., 2024 (52)	700 patients (586 pure DCIS, 114 upstaged)	Mammography	Logistic regression and SVM	Evaluated split-dependent performance bias in DCIS upstaging models; AUC varied from approximately 0.58 to 0.73 across data splits.
Zhu Z. et al., 2019 (49)	131 patients (DCIS only)	MRI	Deep learning + SVM	Predicted occult invasive disease after DCIS diagnosis on MRI; the DL model achieved an AUC of 0.70.
Do et al., 2022 (46)	352 lesions (202 pure DCIS, 150 upstaged)	MRI	Recurrent Residual Convolutional Neural Network using ROIs	Predicted occult invasive upgrade in biopsy-proven DCIS on MRI; the best model achieved an AUC of 0.796 and accuracy of 75.0%. It outperformed 2D CNN (AUC 0.755). Sequential 3D modeling improved results
Mayfield et al., 2024 (48)	154 patients (25 upstaged, 129 pure DCIS)	MRI	CNN-LSTM	Predicted preoperative upgrade of DCIS to invasive cancer on DCE-MRI without segmentation; the CNN-LSTM model achieved a test AUC of 0.73 (VGG16-LSTM) vs 0.62 (ResNet50-LSTM). The study highlights the value of sequential data without need for manual segmentation.
Lee H-J. et al., 2022 (47)	346 patients (349 lesions; 151 upstaged)	MRI	SVM with radiomics	Predicted histologic upgrade of DCIS after surgery on MRI; the SVM radiomics model achieved a test AUC of 0.767, accuracy of 71.4%, and sensitivity 0.733.
Yoon et al., 2024 (4)	420 patients (DCIS only)	Mammography	AI-CAD	Predicted invasive upgrade in biopsy-proven DCIS using AI-CAD abnormality scores; higher scores independently predicted upgrade. Combined model with integration of clinicopathological variables, mammographic features, and AI-CAD scores achieved the highest predictive performance with an AUC of 0.724, outperforming isolated AI-CAD models (AUC of 0.699–0.703) and combination with mammographic features alone (AUC of 0.708).
Park et al., 2022 (50)	644 patients (161 upstaged DCIS)	Multimodal imaging + clinical/pathologic data	Logistic regression and ML models	Predicted invasive upgrade in biopsy-diagnosed DCIS using mammography, ultrasound, and MRI; 161 of 644 cases were underestimated invasive cancers. Model achieved an AUC of 0.66–0.78 and showed no improvement vs. logistic regression. Several predictors of upstaging were identified such as presence of suspicious axillary lymph nodes on ultrasound and high nuclear grade. Factors associated with lower risk included vacuum-assisted biopsy, lesions <2 cm on mammography, and MRI.
Hashiba et al., 2023 (11)	1,387 patients (1,175 COMET-eligible)	Multimodal imaging + clinical/pathologic data	ML	Identified DCIS cases at low risk of surgical upstaging for surveillance selection; Overall surgical upstaging rate from DCIS to invasive cancer was 17%; ML model suggested 56%-87% women for active surveillance compared to 42% based on COMET trial eligibility criteria. Model achieved AUC between 0.52 to 0.58 and sensitivity of 0.89.
Qian et al., 2021 (51)	360 cases (180 pure DCIS, 180 upstaged)	Ultrasound	DL	Predicted postoperative upgrade of DCIS on ultrasound; best of 4 models achieved AUC of 0.802, accuracy of 0.742, specificity of 0.750, and sensitivity of 0.733.
Vy et al., 2022 (53)	420 patients (189 DCIS, 231 MIBC)	Multimodal imaging + clinical/pathologic data	ML	Predicted DCIS vs. minimally invasive breast cancer (MIBC) for therapy guidance. Model combined clinical, mammographic, ultrasonographic, and histopathologic findings and could discriminate DCIS from MIBC with accuracy that paralleled experienced radiologists, the model achieved an AUC of 0.93 and accuracy of 84.0%, specificity of 0.75 and sensitivity of 0.91.
Zhu M. et al., 2022 (54)	568 patients (322 non-microinvasive, 246 microinvasive DCIS)	Ultrasound	Inception-v3 CNN	Identified DCIS with microinvasion on ultrasound; the Inception-v3 model achieved an AUC of 0.803 and accuracy of 76.6%, sensitivity of 0.767, and specificity of 0.765. Performance decreased on external validation from AUC of 0.803 (internal) to 0.685 (external)
Prediction of molecular characteristics and nuclear grade in DCIS				
Zhu M. et al., 2024 (55)	349 patients (pure DCIS)	Ultrasound	DL + ML	Predicted low nuclear grade and receptor status in pure DCIS on ultrasound. Clinical ML models outperformed DL-radiomics models in predicting low nuclear grade (0.710 vs 0.633), ER+ (0.761 vs 0.618), PR+ (0.761 vs 0.755), and HER2+ DCIS lesions (0.723 vs 0.713)
Assessment of intraoperative margins				
Lamb et al., 2022 (56)	650 patients (DCIS only)	Multimodal imaging + clinical/pathologic data	Penalized logistic regression, random forest, multivariable logistic regression	Predicted ipsilateral breast event risk after DCIS treatment using mammography and clinical data; the multivariable model achieved an AUC of 0.75 and outperformed LASSO (0.52) and RF (0.54)
Prediction of recurrence after DCIS treatment				
Li et al., 2018 (13)	66 patients (DCIS not specified)	Multimodal imaging	Multimodal system with ML	Assessed intraoperative breast tumor margins using ultrasound and photoacoustic tomography; the machine learning system achieved 85.5% sensitivity, 90% specificity, and an AUC of 0.93, outperforming an imaging researcher, a breast surgeon and a board‐certified radiologist, in all imaging modality combinations.
Mojahed et al., 2020 (14)	23 patients (3 DCIS)	UHR-OCT	CNN-based classifier	Distinguished cancerous from noncancerous breast tissue for intraoperative margin assessment using OCT; the model achieved 94% accuracy. F1 score for DCIS: 0.65 ± 0.15 (5-fold CV). Lower performance due to small DCIS sample (n=3), but demonstrates feasibility of real-time classification in approximately 0.1 sec/image

AUC, area under the receiver operating characteristic curve; ADH, atypical ductal hyperplasia; AI, artificial intelligence; AI-CAD, AI-assisted computer-aided diagnosis; ADC, apparent diffusion coefficient; CAD, computer-aided detection; CNB, core needle biopsy; CNN, convolutional neural network; COMET, Comparison of Operative to Monitoring and Endocrine Therapy for low-risk DCIS; DCIS, ductal carcinoma in situ; DCE-MRI, dynamic contrast-enhanced magnetic resonance imaging; DL, deep learning; DSS, difference-based, self-supervised; DWI, diffusion-weighted imaging; EHR, electronic health record; ER, estrogen receptor; FFDM, full-field digital mammography; F1 score, harmonic mean of precision and recall; HER2, human epidermal growth factor receptor 2; IBE, ipsilateral breast event; IBC, invasive breast cancer; IDC, invasive ductal carcinoma; LASSO, least absolute shrinkage and selection operator; LDP-BN, latent Dirichlet process Bayesian network; LSTM, long short-term memory; MIBC, microinvasive breast cancer; ML, machine learning; MRI, magnetic resonance imaging; NPV, negative predictive value; NS, not statistically significant; OCT, optical coherence tomography; PAT, photoacoustic tomography; PPV, positive predictive value; PR, progesterone receptor; RF, random forest; ROIs, regions of interest; SOC, standard of care; SVM, support vector machine; train, training dataset; UHR-OCT, ultra-high-resolution optical coherence tomography; US, ultrasound; U-Net, U-shaped convolutional neural network architecture; val, validation dataset; test, testing dataset; internal, internal validation cohort; external, external validation cohort; 3D, three-dimensional; VGG16, Visual Geometry Group 16-layer convolutional neural network; ResNet50 = 50-layer residual neural network.

^**^ Alaeikhanehshir et al. and Wu H. et al. are listed in Grade Classification but also studied Upstaging; hence, their outcomes span multiple domains.

### 3.2 AI in DCIS detection

The integration of AI into mammography and other forms of breast imaging has demonstrated potential for improving the detection of breast cancer overall; its application for DCIS, however, remains inconsistent and inferior to that for invasive cancer.

Among mammography-based systems, the Lunit INSIGHT MMG model (Version 2019, Lunit Inc., Seoul, South Korea), an AI-CAD system trained for full-field digital mammography, outperformed traditional mammography interpretation in detecting DCIS with a sensitivity of 100% compared with 88.9% (P < 0.0001, McNemar test) (17). However, the number of DCIS cases (n = 9) was very small, and the perfect sensitivity of the AI model likely inflated the McNemar test significance, limiting the generalizability.

In contrast, Waugh and colleagues found that radiologists outperformed Transpara CNN (version 1.7.0, ScreenPoint Medical, Netherlands), an AI model using DL algorithms within a CNN, in overall detection sensitivity by 100% to 94% respectively (P = 0.05). When the results were evaluated for DCIS detection alone, the AI model showed a lower detection sensitivity (92.3%) compared to the radiologists (100%) (18). Again, however, the DCIS sample size (n = 13) was small.

Using a substantially larger mammographically detected and histologically confirmed dataset, Weigel and colleagues evaluated Transpara CNN (version 1.7.0) in 634 patients with 644 calcification-related lesions, including 151 DCIS lesions and 44 with invasive breast cancer. They reported a significantly higher false negative rate for detecting DCIS (8.6%; 13/151) compared to invasive cancers (2.3%;1/44 cases) (19). Their analysis also revealed substantial overlap between AI-generated malignancy scores for DCIS (median:74; interquartile range (IQR): 52–84) and benign lesions (median: 61; IQR: 45–74), limiting the model’s ability to discriminate between them (19). These findings align with broader evidence that DCIS imaging signatures are subtle and heterogeneous. Similarly, Lee and co-authors, after analyzing 896 patients with 930 cancerous breasts, found that a high proportion (52/192; 28.9%) of DCIS-positive breasts were recorded as negative using AI-based computer-assisted diagnosis (AI-CAD), compared to only 13.6% of the high-scored cancers being classified as DCIS, suggesting that DCIS is more likely than invasive cancers to be missed by current systems (20). Collectively, these mammography-based studies suggest that although AI systems may approach radiologist-level performance in some contexts, DCIS remains more likely to be missed than invasive disease.

Beyond mammography, several studies have evaluated ultrasound-based AI systems, where evidence for DCIS detection is even more limited. Xiao and colleagues assessed a DL framework-based CAD (DL-CAD) system on 448 various breast lesions including 29 DCIS lesions (21). DL-CAD performed significantly better than a resident as well as an experienced radiologist in terms of diagnostic performance and accuracy when identifying rare or complex benign lesions; it correctly classified 88.1% of fibroadenomas (119/135) and 71.4% of adenosis (25/35). However, its accuracy dropped below that of the two radiologists in specifically distinguishing DCIS; DL-CAD had a diagnostic accuracy of 72.4% (21/29) in identifying DCIS, which was lower compared to 96.6% for the experienced radiologist (28/29) and 86.2% for the resident (25/29). While these differences were not statistically significant, the findings highlight the ongoing difficulty AI systems face in reliably identifying DCIS. Nevertheless, the data suggest that the use of DL-CAD would help decrease the number of unnecessary breast biopsies overall (21).

Similarly, AI-SONIC Breast intelligent assisted diagnosis system (Zhejiang Deshan Yunxing Company, China), which employs a DL framework and CNNs to analyze ultrasound images of breast masses, was evaluated in 196 patients with 202 masses, classified as Breast Imaging Reporting and Data System (BI-RADS) 3-5, and included 10 DCIS cases (22). When compared with three radiologists with 23, 10 and 3 years of experience, AI-SONIC improved the accuracy, sensitivity, and negative predictive value (NPV) for BI-RADS 4a and positive predictive value (PPV) for 4b lesions. It also increased the diagnostic sensitivity for DCIS from 70% at baseline for all three radiologists, irrespective of experience, to 80% in all cases; however, this improvement was not statistically significant. It must also be noted that AI enhanced the detection sensitivity of invasive breast cancer more than it did for DCIS. These findings suggest that while AI-assisted interpretation may improve accuracy for certain benign or invasive lesions, improvements for DCIS remain modest and statistically nonsignificant.

Overall, the observed variability in the detection of DCIS by AI-based applications stems from several factors including very small numbers of DCIS cases within much larger pools of other breast cancer types, heterogeneity in imaging modalities, and the design of AI systems that do not explicitly target DCIS as a primary diagnostic category (4, 7, 17, 19). Algorithmic refinements that can better capture the subtle imaging features of DCIS as well as more robust, large-scale, and DCIS-specific training datasets are needed to determine how well the models would perform in broader clinical practice.

### 3.3 AI in DCIS classification and risk stratification

Unlike detection studies, which focus on identifying the presence of malignancy, classification-based AI models aim to distinguish DCIS from other lesions or to stratify DCIS into biologically or clinically meaningful subgroups. These applications are particularly relevant for reducing overtreatment and guiding surgical or surveillance decisions.

#### 3.3.1 Discriminating DCIS from other lesions

Differentiating DCIS from other cancer types, such as IDC, ADH, fibroadenoma, and fibromatosis, is challenging due to overlapping imaging features and histological similarities, especially in early or borderline lesions. These conditions can mimic each other on mammograms and biopsies, complicating accurate diagnosis and treatment planning. Multimodal assessment involving imaging, pathology, clinical findings, and, in some cases, surgical excision for definitive classification, is needed. This section outlines the current status of AI in helping delineate these malignancies from each other.

##### 3.3.1.1 Invasive ductal carcinoma

Distinguishing DCIS from IDC is challenging in breast cancer diagnostics, as both may present with calcifications and ductal changes in imaging. Conventional imaging modalities often struggle to differentiate these entities that overlap in radiologic features, particularly in mammography and MRI, leading to both under- and over-treatment in clinical practice. While IDC requires prompt intervention due to its metastatic potential, DCIS represents a heterogeneous, non-obligate precursor lesion for which overtreatment remains a persistent concern (23, 24). As precision oncology increasingly emphasizes tailored therapeutic strategies, the development of AI tools capable of reliably distinguishing DCIS from invasive disease has become an important research priority. AI offers the potential to extract subtle imaging features beyond human perception, reduce inter-observer variability, and integrate multimodal imaging modalities such as diffusion-weighted MRI, dynamic contrast-enhanced MRI, mammography, and ultrasound, along with clinical information, thereby improving diagnostic accuracy and supporting more individualized care. Successfully differentiating DCIS from IDC would have direct implications for patient management, treatment selection and intensity, and long-term outcomes including prognosis and quality of life.

Four studies (25–28) collectively demonstrate that AI can enhance diagnostic accuracy, improve workflow efficiency, and incorporate demographic or contextual information to refine predictions. Yin and colleagues performed AI-based differentiation of DCIS and IDC on a population of 802 patients (347 DCIS cases, 43.3%) using diffusion-weighted imaging (DWI) (27). Consistent with prior radiologic evidence that IDC exhibits lower apparent diffusion coefficient (ADC) values than DCIS (P < 0.001) due to higher cellular density and disrupted tissue microstructure (29), they developed CNNs trained on ADC maps. Their CNNs significantly outperformed traditional mean-ADC measurements obtained through manual region-of-interest (ROI) placement on the internal test set (P = 0.001), while showing a non-significant trend toward improved performance on the external test set (P = 0.096). The CNN-based approach also improved multiple diagnostic metrics, including AUC, sensitivity, specificity, positive predictive value (PPV), negative predictive value (NPV), and accuracy while reducing inter-observer variability. Beyond accuracy, the model reduced per-lesion processing time from 3–8 minutes for manual ADC measurement to approximately one second per lesion, demonstrating the potential for meaningful workflow integration. These findings highlight the value of AI-enhanced DWI in capturing microstructural differences between DCIS and IDC that may be difficult to quantify manually.

Jayender and colleagues developed a Statistical Learning Algorithm for Tumor Segmentation (SLATS) combining hidden Markov models (HMMs), Fast Fourier Transform (FFT), Short-Time Fourier Transform (STFT), and fuzzy c-means clustering to analyze dynamic contrast-enhanced MRI (DCE-MRI) (25). In a small but well-characterized cohort of 41 cases comprising 19 DCIS and 22 IDC cases, the SLATS algorithm achieved high accuracy (92.6%) and sensitivity (100%) in detecting IDC when using composite vectors, outperforming the CADstream system (Merge Healthcare Inc., Wisconsin, USA), which missed 9% of invasive tumors. Although accuracy for DCIS was lower (79.3%), likely due to weaker tumor enhancement patterns typical of *in situ* lesions, SLATS detected 26.3% of DCIS cases missed by CADstream. SLATS also demonstrated strong noise resistance and consistent performance across DCE-MRI datasets with varying time points. These results suggest that AI-based temporal modeling of contrast kinetics may improve detection of subtle enhancement patterns associated with DCIS; however, larger studies are needed to validate these findings.

Nassif and colleagues addressed a different but clinically relevant challenge: the tendency for mammography to overdiagnose DCIS in older women aged 65 and above (26). They integrated inductive logic programming (ILP)–derived age-specific rules, filtered through performance thresholds (recall >10%, precision >60% for older women), into a mammography-based Logical Differential Prediction-Bayes Net (LDP-BN). They then compared a baseline Bayes Net model that incorporated a Tree Augmented Naive Bayes (TAN) structure with mammography features such as BI-RADS categories but lacked age-specific rules to the LDP-BN model. Two cohorts were employed: a cohort of older women aged 65 years and above comprising 101 invasive cancer and 112 DCIS mammograms, and a second cohort of younger women less than 50 years old containing 264 invasive and 110 DCIS mammograms. The LDP-BN achieved an AUC of 0.8911, significantly outperforming the baseline Bayes Net model (AUC = 0.8304; P < 0.0001) in distinguishing DCIS from IDC in older women. Their work underscores the importance of demographic-aware modeling and suggests that AI can incorporate contextual clinical information such as age-related differences in tumor biology to refine diagnostic predictions.

Zhang and colleagues developed an ultrasound radiomics model that incorporated a combination of intratumoral and peritumoral features for differentiating IDC from DCIS and validated this model using a cohort of 629 patients that were divided into a training group (363 cases), a validation group (91 cases), and an external test group (175 cases) (28). They determined that optimal performance was achieved when intratumoral features were combined with an 8 mm peritumoral region, yielding a 9.7% AUC increase in the validation group and a 6.4% AUC increase in the test group, compared to the intratumoral-only model (P = 0.031 and P = 0.005, respectively). Their study demonstrated the utility of integrating data from surrounding tissues for AI-based differentiation of DCIS and IDC using ultrasound.

Collectively, these studies demonstrate AI’s potential in enhancing DCIS vs. IDC differentiation across imaging modalities, with ADC-based CNN offering high accuracy and efficiency in DWI compared to the traditional, manual ADC model and SLATS excelling in DCE-MRI for IDC detection. For mammography, the LDP-BN underscores the importance of demographic-aware modeling. Future research should validate these approaches in larger, multi-institutional cohorts to ensure generalizability.

##### 3.3.1.2 Atypical ductal hyperplasia

ADH is a non-obligate precursor to DCIS or invasive breast cancer characterized by WHO quantitative criteria of size ≤2 mm and involvement of ≤2 membrane-bound spaces, exhibiting features of partial atypia but lacking the full cytological and architectural features of DCIS. Thus, ADH shares cytological features with low-grade DCIS. Approximately 70–90% of ADH cases remain benign upon surgical excision, carrying a relatively lower 10–30% risk of upgrade to malignancy such as DCIS or invasive cancer compared to that of DCIS, which carries a 20–50% risk of post-surgical upstaging to invasive breast cancer (1). The use of AI can overcome sampling constraints of traditional biopsies by analyzing entire mammographic regions. Accurate differentiation of ADH from DCIS could address overtreatment concerns, potentially sparing ADH patients from surgery by recommending adherence to emerging active surveillance protocols (15, 30).

Ha and colleagues developed and evaluated a CNN to differentiate ADH from DCIS using a retrospective dataset of 298 unique mammographic images from 149 women (134 images from 67 ADH patients and 164 images from 82 DCIS patients) (30). They reported an overall diagnostic accuracy of 86.7%, an AUC of 0.86, sensitivity of 84.6%, and specificity of 88.2%, demonstrating the effectiveness of CNN algorithms in this context (30).

Mutasa and colleagues prospectively validated a CNN model using a new dataset composed of 280 mammographic images from 140 patients, with 61 patients diagnosed with ADH and 79 diagnosed with DCIS (15). They achieved an accuracy of 80.7%, specificity of 93.7%, sensitivity of 63.9%, and an AUC of 0.9. The high specificity suggests that the model was effective in minimizing the number of ADH cases misclassified as DCIS; however, the lower sensitivity indicates that a substantial proportion of DCIS cases may still be misclassified as ADH. Notably, the ADH cohort was statistically significantly younger, with a mean age of 56.1 ± 12.2 years compared to the DCIS cohort (61.9 ± 11.1 years; P = 0.01), raising the possibility that model performance may have been influenced in part by age-related cohort differences rather than imaging features alone. This represents an important potential confounder and highlights the need for larger, multi-institutional validation datasets with careful adjustments for demographic and clinical variables. Both studies (15, 30) proposed that validation with larger, multi-institutional datasets are needed to improve model reliability and assess generalizability.

Although current mammography-based CNN models designed for differentiating ADH from DCIS are not yet viable substitutes for core needle biopsies (CNB), their complementary use may enhance diagnostic accuracy and reduce unnecessary surgeries. The strength of CNN use in this task lies in its ability to perform comprehensive imaging analysis that can alleviate biopsy sampling constraints and enhance stratification of mammographically low-risk ADH patients into active surveillance trials.

##### 3.3.1.3 Fibroadenoma

Fibroadenomas (FA), although benign, can mimic DCIS on mammograms, particularly when they are calcified. Yin and colleagues investigated the use of ultrasound radiomics-based AI in differentiating DCIS from FA, testing several feature engineering-based ML (FEML) models as well as DL models on a prospective test cohort of 139 DCIS and 181 FA patients from a total cohort of 461 DCIS and 651 FA patients (31). FEML models achieved a maximum AUC of 0.7935 on the independent prospective test cohort, while the highest-performing DL model achieved an AUC of 0.7656. They also developed a novel difference-based self-supervised (DSS) learning approach that required only FA samples for training and highlighted grayscale distribution differences between DCIS and FA, leveraging reconstructed images for sample classification. The DSS model achieved an AUC of 0.8172, outperforming conventional models.

##### 3.3.1.4 Fibromatosis

Fibromatoses are rare and benign; they can resemble invasive carcinoma both histologically and radiologically. Jiang and colleagues developed a clinical-ultrasound multimodal ML model using logistic regression that combined clinical data, ultrasound features, and radiomics to differentiate between DCIS and breast fibromatosis (32). They trained six ML algorithms on radiomics features to extract hidden features such as tumor shape and texture from ultrasound images taken from a cohort of 306 patients (198 DCIS and 108 breast fibromatosis patients) that was further sub-divided into a development cohort (n = 184) and a validation cohort (n = 122).They found that, of the six algorithms tested, the logistic regression-based model performed the best, achieving an AUC of 0.947–0.991. Further integration of clinical factors (e.g., age, BI-RADS grade) into a combined clinical and radiomics model improved prediction AUC to 0.983–0.996, suggesting that integration of relevant clinical features can enhance radiomic model performance in differentiating DCIS from fibromatosis and helping clinicians reduce overtreatment.

Beyond distinguishing DCIS from other benign or malignant entities, recent work has explored whether AI can further stratify DCIS itself into risk categories based on imaging and multimodal data.

#### 3.3.2 DCIS grade classification based on imaging modality

DCIS grade classification remains subjective, with only moderate inter-observer agreement between experts. Since AI has the potential to extract and analyze subtle morphological and radiomic features that may be missed by humans, several groups have investigated the use of AI in non-invasively stratifying DCIS into clinically meaningful tiers (1, 5).

Wu and colleagues evaluated a DL multimodal information model (Multimodal-DCIS-Net) which integrated the use of ultrasound, mammography, CNB pathology, and clinical variables for classifying DCIS into 3 categories: low-grade, intermediate-to-high-grade, and upstaged disease in a cohort of 733 patients (1). The model achieved accuracies of 0.752–0.766 in the three-classification task and 0.751-0.780 in discriminating pure DCIS from upstaged DCIS, compared to 0.511 and 0.681 respectively for an expert radiologist. These findings suggest that multimodal integration substantially enhances predictive performance.

In contrast, Alaeikhanehshir and colleagues evaluated a mammography-only U-Net-based CNN in 464 patients to differentiate low-risk (grade I/II) from high-risk (grade III) DCIS (5). The model achieved an AUC of 0.72, PPV of 40.3%, and NPV of 90.9%. The lower performance relative to multimodal approaches underscores the limitations of single-modality imaging when attempting to predict DCIS biological behavior.

Recently, a radiomic ensemble ML model based on Elastic Net, ranger, and Generalized Linear Models with Boosting was developed by integrating radiomic scores from 241 cases with ultrasound, mammography images and pre-operative clinical data to predict low nuclear grade DCIS (33). This model achieved an AUC of 0.92 on the validation set, significantly outperforming another model that only used clinical data (AUC = 0.86). It also improved discrimination and risk stratification in DCIS. Together, these studies suggest that multimodal and radiomic-enhanced approaches may improve non-invasive DCIS risk stratification, with potential implications for surveillance strategies, surgical planning, and adjuvant therapy selection.

MRI-based AI models have also shown promise for general breast cancer classification but continue to underperform for DCIS. Liu and colleagues developed an unenhanced radiomics-only model that used ML on non-gadolinium-based T2-weighted (T2-W) and diffusion-weighted (DW) MRI to distinguish between benign and malignant breast lesions in a total cohort of 1760 patients, including 61 DCIS cases (34). The model achieved an AUC of 0.893 for the training cohort and 0.848 for the validation cohort (34). Importantly, it demonstrated comparable diagnostic accuracy to a combined model based on T2W, DW, and the first contrast-enhanced T1-weighted sequence; AUC differences across all cohorts were not significant, demonstrating promising potential for screening for breast cancer using MRI without the need for contrast, enabling potential cost savings, and serving as a safer alternative for patients who have contraindications to the use of contrast agents. However, DCIS remained the model’s weakest area with a sensitivity of 0.677, substantially lower than for invasive ductal carcinoma (0.92) and invasive mammary carcinoma (0.88). This disparity reinforces the broader pattern observed across modalities: AI systems consistently perform better for mass-forming invasive cancers than for DCIS, whose imaging features are more subtle and heterogeneous.

#### 3.3.3 AI in clinical-decision support classification

Another clinically relevant application of AI classification, beyond biological grading, is refining BI-RADS categorization and reducing unnecessary biopsies (35, 36).

Barros and colleagues developed a CNN-based model to classify breast lesions using digital mammography images and linked electronic health records (35). The model achieved overall AUCs of 0.80 and 0.88 in cohorts of American (n = 2,336) and Israeli (n = 3,017) patients respectively, for distinguishing malignancy. Although DCIS-specific performance was lower in both the American (11.3% DCIS; AUC = 0.74) and Israeli cohorts (18% DCIS; AUC = 0.76), the system maintained an overall sensitivity of 99% and could prevent 13% of unnecessary biopsies while missing only 1.3% of malignancies, including DCIS. It, therefore, demonstrated the potential to reduce the number of invasive procedures (35).

Similarly, Liu and co-authors showed that AI models with CNNs could help in accurately sub-classifying lesions categorized on the basis of BI-RADS (36). In the BI-RADS mammographic assessment system, lesions are categorized as BI-RADS 4 when they are suspected to be malignant and biopsy is recommended. BI-RADS 4 lesions can be further sub-divided into 4A, 4B, and 4C categories based on low, moderate and high suspicions of malignancy respectively. When Liu and colleagues tested their model with a cohort of 847 patients, of which 200 patients were diagnosed with DCIS, the model achieved an AUC of 0.73 when differentiating between benign lesions and DCIS. It correctly identified 25% of benign BI-RADS 4 lesions while maintaining a 95% sensitivity for DCIS detection, supporting a role for AI in reducing unnecessary invasive procedures. The model also showed improved performance in distinguishing benign lesions from atypia (AUC = 0.70) and benign lesions from invasive tumors (AUC = 0.72) (36).

Similar trends were observed in ultrasound-based systems such as DL-CAD (evaluated previously in subsection 3.2), which demonstrated improved classification of benign lesions and potential reduction in unnecessary biopsies, although DCIS-specific accuracy remained lower than that of radiologists (21).

### 3.4 AI in risk assessment

Conventional risk assessment tools for breast cancer, which rely on demographic, reproductive, and family history variables, have demonstrated moderate predictive accuracy in identifying women who may develop breast cancer (37, 38); it is now recognized that breast cancer risk is multifactorial and involves complex interplay of genetic susceptibility, hormonal exposures, lifestyle behaviors, and intrinsic breast tissue characteristics (39, 40). Accordingly, a more comprehensive approach that combines these factors as well as emerging biomarkers and imaging-based metrics to enhance individualized risk stratification needs to be adopted. Such an integrated model would enable optimization of screening parameters and provide better prevention strategies to reduce morbidity and mortality associated with breast cancer. With its capacity to rapidly process big data and its potential to extract subtle imaging biomarkers that may precede clinical diagnosis, AI is expected to play a major role in risk assessment. However, current evidence suggests that the benefits of AI are uneven across breast cancer subtypes, with consistently lower predictive accuracy for DCIS compared with invasive disease.

This distinction is illustrated by Lee and colleagues (6). Using an AI-CAD system, Lunit INSIGHT MMG (version 1.1.0.0), they retrospectively evaluated abnormality score trajectories of 487 mammograms from 126 women who later developed breast cancer; 30 of these women developed DCIS. Overall, abnormality scores of breasts later diagnosed with cancer increased significantly with a slope of +0.6 in 6 months (P < 0.001) as compared to no meaningful change in contralateral normal breasts (+0.03; P = 0.776). However, when the data was mined for elucidating differences between invasive breast cancer vs. DCIS, invasive cancers showed a marked rise in slope (+1.4 per 6 months; P = 0.002) whereas no significant temporal trend was noted for DCIS (P = 0.771). These findings demonstrate the potential of AI-CAD in early detection of invasive cancer while highlighting the need for further research in detecting DCIS-specific risk patterns (6).

The difficulties underlying DCIS prediction were also reflected by Damiani and colleagues (41). These investigators used Mirai (version 0.3.1, https://github.com/yala/Mirai), a DL AI model that analyzes mammograms to generate a personalized risk score of developing breast cancer in five years. They achieved moderate accuracy (AUC = 0.68) in predicting breast cancer risk within three years of a negative screening exam. Notably, there was no significant difference (P = 0.085) in predictive performance between interval cancers, which are those diagnosed between regular screenings, often due to emerging symptoms, and screen-detected cancers, which are identified during routine mammography; the AUCs were 0.69 and 0.67 respectively. Similarly, the model showed comparable performance in predicting invasive cancers (AUC 0.68) and DCIS (AUC = 0.66). However, the model was significantly more effective (P = 0.037) at predicting advanced cancer risk (≥stage II) compared to earlier-stage cancer (<stage II) with AUCs of 0.72 and 0.66 respectively, suggesting that current AI risk models may be more attuned to biologically aggressive disease. The relatively lower performance for DCIS again reinforces the need for algorithms capable of capturing the more subtle or heterogeneous imaging signatures associated with *in situ* lesions (41).

Additional insight into the relationship between AI detection and risk prediction for DCIS was provided by a paired design study that evaluated the performance of four AI algorithms, including Mirai (Massachusetts, USA) as well as GMIC, NY-H, and NY developed by New York University (New York, USA), across 3,386 matched mammogram pairs that included 420 DCIS cases (42). Specifically, the study directly compared algorithm performance in detection and risk assessment using mammograms from the same women taken at two time points: at cancer diagnosis (“detection”) and at their previous screening visit three years prior (“risk assessment”). The authors found that models with superior diagnostic performance also tended to perform better in risk assessment. Mirai achieved the highest AUC for risk assessment (0.67) and detection (0.89) overall. However, across all algorithms, risk prediction for DCIS remained consistently lower than for invasive cancers. While DCIS detection AUCs ranged from 0.88 (Mirai) to 0.93 (GMIC), risk AUCs were notably reduced for both Mirai (0.66) and GMIC (0.66), indicating that even high-performing diagnostic models struggle to anticipate DCIS development years in advance. While these diagnostic models may be repurposed for risk stratification, comparably reduced performance should be expected for DCIS at this time.

In addition to risk prediction, AI models have also been explored for monitoring risk modification. Manley and colleagues investigated the use of a CNN to predict breast cancer risk before and after chemopreventative therapy based on mammographic images as opposed to conventional risk analysis models and human-chosen feature analysis (43). Among the 541 patients included, 206 (38%) had a diagnosis of DCIS, 215 (39.7%) had atypical hyperplasia, and 120 (22.3%) had lobular carcinoma *in situ*. The authors noted that a significantly higher proportion of patients who underwent chemoprevention treatment experienced a decrease in their CNN-based breast cancer risk score compared to those who did not receive treatment (33.7% vs. 22.9%; P < 0.01). Moreover, significantly fewer women who underwent chemoprevention treatment had an increase in their CNN-based breast cancer score compared to those who did not undergo treatment (11.4% vs. 20.2%; P < 0.01). Multivariate analysis revealed that treated women were 1.29 times more likely to see risk reduction, confirming that chemoprevention negatively correlated with risk score increases (P = 0.02). These findings highlight the utility of a CNN-based mammographic risk model to evaluate the efficacy of existing treatments in certain patient populations, perform personalized risk assessment, and potentially improve compliance with treatment strategies.

Collectively, AI-enhanced mammographic models demonstrate promising capability for longitudinal risk assessment and early signal detection, particularly for invasive and advanced-stage cancers. However, across multiple independent cohorts and algorithms, predictive performance for DCIS remains consistently lower. This pattern mirrors findings in detection and classification studies, suggesting that current AI systems may preferentially capture imaging features associated with biological aggressiveness rather than the more subtle and heterogeneous characteristics of *in situ* disease. Future progress will likely require DCIS-specific imaging biomarkers, larger longitudinal datasets enriched for *in situ* lesions, and models explicitly optimized to detect pre-invasive risk trajectories.

### 3.5 AI in upstaging

As a non-obligate preinvasive precursor, DCIS presents a variable 20–50% risk of postsurgical upstaging to invasive breast cancer (1), posing challenges such as difficult risk stratification and overtreatment (2). Accurate preoperative prediction of upstaging is, therefore, critical for guiding clinical decisions regarding sentinel lymph node biopsy, surgical extent, and eligibility for active surveillance protocols and appropriate management.

Our search yielded 13 studies that examined the use of AI in the prediction of DCIS pre-surgical upstaging. These are categorized by imaging modality to allow for more effective intra- and inter-modality synthesis: four studies each involving mammography (5, 12, 44, 45), MRI (46–49), and multimodal integration (1, 4, 11, 50), and one using ultrasound (51). This framework allows for direct comparison of model predictive performance, methodological approaches, clinical applicability, and limitations across modalities.

#### 3.5.1 Mammography

Mammography-based AI models for DCIS upstaging prediction have generally demonstrated moderate discriminatory performance, with AUCs ranging from approximately 0.68 to 0.76, though sensitivity–specificity trade-offs vary considerably across studies.

Mutasa and colleagues employed a CNN-based algorithm to predict pre-surgical upstaging of DCIS using mammographic images (44). The study included a total of 123 patients, of which 82 patients were diagnosed with pure DCIS and 41 DCIS patients upstaged to invasive disease following a stereotactic biopsy diagnosis. Compared to the COMET and LORIS clinical trials, which report accuracies of 40% and 39% respectively, the CNN performed at a reported overall diagnostic accuracy of 74.6% with an AUC of 0.71 in distinguishing pure DCIS patients. Notably, specificity was high (91.6%) but sensitivity was low (49.4%), indicating stronger performance in correctly identifying pure DCIS than in detecting occult invasion. While the high specificity may reduce overtreatment, the relatively low sensitivity raises concern regarding missed invasive disease.

Using the largest DCIS mammography dataset at the time, consisting of 700 women with DCIS, of whom 114 (16.3%) were upstaged to invasive cancer at surgery, Hou and colleagues developed a U-Net-based CNN combined with logistic regression and radiomic feature selection for the predicting DCIS upstaging (52). Lesions with well-established clinical features indicative of upstaging, such as palpability, asymmetry, architectural distortion, and mass, were excluded by employing a selection of 109 radiomic features and 4 clinical features. The best-performing model, which combined radiomic and clinical features without selection, achieved an AUC of 0.71; at a fixed high sensitivity of 90%, the specificity of the model was 22%, and NPV was 92%. At a fixed high specificity of 90%, sensitivity was 37% and PPV was 41%. However, this performance improvement was not statistically significant compared to the model that combined radiomic and clinical features with selection (AUC 0.68) or the radiomics-only model with selection (AUC 0.69). The latter two models were notably identical, as 11 radiomic features were selected by logistic regression with L2 regularization; none of the four clinical features (progesterone receptor, estrogen receptor, nuclear grade, or age) were employed, indicating that including these clinical features did not improve predictive performance, suggesting that imaging-derived features may capture much of the relevant predictive signal (52).

Shi and colleagues investigated whether a CNN-based model pre-trained on 20 million non-medical images (e.g., animals, plants, and instruments) to learn generic visual hierarchies (edges, textures, and shapes) could extract meaningful patterns (deep features) from mammography to predict pre-surgical DCIS upstaging without additional domain-specific learning (45). Using a sample of 99 DCIS patients, which included 25 patients upstaged on the basis of CNB, the general-purpose CNN was compared to a handcrafted, computer vision (CV) model, engineered explicitly using prior domain knowledge of mammographic calcifications with integration of shape features such as microcalcification morphology, topological features such as cluster graphs, and textural features such as gray level matrices. The general-purpose CNN model performed comparably to the handcrafted CV features model (AUC of 0.70 vs. 0.68, respectively; P = 0.074), demonstrating that domain-specific learning was not required for effective transferability and potential for generalization to medical tasks. Importantly, the middle layer (layer 3) of the general CNN model achieved the best performance with an AUC of 0.66 compared to both shallower layers (AUC = 0.58 for layer 0) and deeper layers (AUC = 0.61 for layer 4), indicating that mid-level CNN features allow for a better balance between generalizability and task specificity in radiomic prediction. In both the general CNN model and CV model, feature selection significantly improved performance (P < 0.0001), with the CNN’s AUC improving from 0.64 to 0.70 and the CV model’s AUC improving from 0.64 to 0.68, highlighting that many of the extracted features were redundant or noisy (45).

Alaeikhanehshir and colleagues, while primarily developing a CNN to distinguish low-risk (grade I/II) from high-risk (grade III) DCIS, also evaluated its ability to predict pre-surgical upstaging among biopsy-diagnosed low-risk cases using mammographic features (5). In a cohort of 464 DCIS patients distributed into an 80/20 training/testing split with five-fold cross-validation, the model achieved an AUC of 0.76 with a PPV of 80.0% and NPV of 83.9%. This represents the highest reported AUC among mammography-only models in this subgroup and suggests that grade-discriminative imaging features may overlap with those predictive of invasive potential.

Collectively, mammography-based AI models demonstrate reproducible but moderate predictive accuracy for DCIS upstaging. Performance typically clusters around AUC values of 0.70 to 0.76, with models often favoring either high specificity (minimizing overtreatment) or high sensitivity (minimizing missed invasion) but rarely optimizing both simultaneously. These findings suggest that mammographic radiomic features capture relevant signals of invasive potential yet remain insufficient as standalone predictors for definitive surgical decision-making.

#### 3.5.2 Magnetic resonance imaging

ML and DL models have been applied to breast MRI for predicting pre-surgical DCIS upstaging, employing a range of architectures, such as two-dimensional (2D), three-dimensional (3D), time-independent and time-dependent analyses, and both radiomic feature–based and end-to-end deep learning pipelines. Compared with mammography-based models, MRI approaches generally demonstrate slightly higher discriminatory performance, though methodological heterogeneity and limited cohort sizes constrain generalizability.

Lee and colleagues developed a radiomics-based ML using a support vector machine (SVM) trained on features extracted from breast DCE-MRI to predict the likelihood of pre-surgical DCIS being upstaged to invasive cancer (47). Their study included a cohort of 349 lesions from 346 female patients, of which 151 cases (43.3%) were ultimately upstaged. The model achieved a sensitivity of 0.733, a specificity of 0.7, an accuracy of 0.714, and an AUC of 0.767. Four-fold cross-validation demonstrated a mean validation accuracy of 0.724 and an AUC of 0.742, further supporting the reliability of the results. Although the use of semi-automatic segmentation was time-consuming, intratumoral ROI reproducibility was high (ICC 0.937 ± 0.072), indicating strong interobserver consistency. This study represents one of the higher-performing MRI radiomics models for upstaging prediction.

Zhu and colleagues applied DL algorithms to DCE-fat-saturated T1-weighted MRI sequences of 131 patients with CNB-confirmed DCIS (49). The MRI scans were analyzed using two approaches: one model based on transfer learning with a pre-trained GoogleNet and another model that was pre-trained on ImageNet to extract deep features from the images and used a polynomial SVM kernel to predict upstaging. The deep features model achieved an AUC of 0.70 compared to an AUC of 0.68 for the transfer learning model. Although the integration of DL for feature extraction is promising, the SVM relied on manual or semi-automatic segmentation and handcrafted radiomic features, and the study sample size was relatively small (n = 131). Notably, the transfer learning approach achieved an AUC of 0.96 after 10 epochs on the training dataset; it likely suffered from overfitting, indicating that it may have learned the training data too well, including the outliers and noise, leading to poor generalization on new data. The authors concluded that larger, more diverse multi-institutional datasets may be needed to improve the accuracy and reliability of transfer learning models (49).

Several investigators have explored whether incorporating spatial or temporal context improves predictive performance. Do and colleagues evaluated the use of long short-term memory layers (LSTMs) for analyzing sequential relationships across multiple MRI slices, essentially enabling 3D volumetric interpretation (46). They analyzed 352 DCIS lesions confirmed by CNB (202 pure DCIS and 150 upstaged DCIS) using three DL models, including a Detection-Transfer Recurrent Residual Convolutional Neural Network (D-T RRCNN) model that considered 3D MRI slices as a sequence of 2D images and automated quarter-section breast MRI localization, a Recurrent Residual Convolutional Neural Network (RRCNN) model that employed explicit tumor localization via manual ROIs, and a standard CNN with ROIs that was only able to analyze single 2D slices. The RRCNN with ROIs model achieved the highest performance in terms of sensitivity, specificity, and accuracy compared to the other models which was statistically significant, highlighting better performance of sequential models using multiple MRI slices, allowing for the analysis of volumetric data across adjacent slices when compared to single-slice models. Thus, sequential slice modeling better captures tumor heterogeneity and invasive characteristics than single-slice analysis.

Mayfield and colleagues extended this concept to temporal modeling by analyzing multiphase DCE-MRI sequences using hybrid CNN–LSTM architectures. They evaluated time-dependent DCE-MRI DL models that analyzed temporal changes in contrast uptake in breast MRI scans to predict whether DCIS would be upgraded to invasive cancer (48). They compared two hybrid models, VGG16-LSTM, which combined a deep CNN (VGG16) known for its strong image classification performance, with an LSTM, and ResNet50-LSTM, a deep CNN model designed to mitigate vanishing gradient problems. The models were evaluated using 154 cases, of which 129 were pure DCIS and 25 were upgraded to DCIS. VGG16-LSTM model with multiphase contrast performed the best overall, achieving a multiphase test AUC of 0.73 compared to the ResNet50-LSTM, which achieved an AUC of 0.62, demonstrating significantly better performance (P = 0.008). In addition, when comparing single-point model performance, VGG16 still significantly outperformed ResNet50 (P = 0.04) with a multiphase test AUC of 0.67 compared to 0.59, respectively. The performance of the time-dependent VGG16-LSTM model with an AUC of 0.73 when compared to the single-point-in-time VGG16 model, which achieved an AUC of 0.67, demonstrates that the time-dependent (sequential deep learning algorithms) using preoperative DCE MRI significantly improved the prediction of DCIS surgical upstaging (P = 0.04). The results also show that modeling contrast enhancement kinetics improves prediction accuracy. Additionally, sequential models reduce reliance on manual segmentation compared with static ROI-based approaches, potentially decreasing inter-operator variability.

Overall, MRI-based AI models for DCIS upstaging prediction typically achieve AUCs ranging from approximately 0.70 to 0.77, modestly higher than most mammography-only models. Incorporation of volumetric (3D) or temporal (multiphase DCE) information appears to improve predictive performance compared with static 2D approaches, suggesting that invasive potential may be encoded in spatial heterogeneity and enhancement dynamics. However, many studies remain limited by relatively small single-institution cohorts, reliance on manual segmentation, and lack of external validation, restricting immediate clinical translation (46, 48).

#### 3.5.3 Ultrasound

Ultrasound has limited accuracy in predicting DCIS upstaging due to its lower sensitivity for detecting microinvasion, limited spatial resolution, poor visualization of microcalcifications as well as subtle or non-mass lesions, and operator dependency, leading to inconsistent lesion characterization.

Qian and co-workers trained and tested four CNN-based DL models that either incorporated a ResNet architecture characterized by residual or skip connections or the simpler VGGNet architecture, which stacks multiple 3x3 convolution layers followed by max pooling; these included ResNet-b0, ResNet-b1, ResNet-b2, and VGGNet. The authors analyzed 2D ultrasound images from 360 patients with DCIS diagnosed by CNB (51). Of these, the ResNet-b1 model performed the best overall, achieving an AUC of 0.802, sensitivity of 0.733, specificity of 0.750, and accuracy of 0.742 in the validation set (n = 120). A 3-fold cross-validation for ResNet-b1 yielded AUCs of 0.767, 0.808, and 0.760, confirming the model’s robustness. In contrast, the VGGNet-based model showed overfitting, with a significant performance gap between training and validation sets (AUCs of 0.992 and 0.724, respectively).

Importantly, the AUC of 0.802 is higher than that reported in several mammography-only and MRI-only studies; thus optimized, ultrasound-based DL models can achieve competitive performance. However, ultrasound’s intrinsic limitations in detecting subtle invasive foci and calcification-associated DCIS require cautious interpretation and external validation.

#### 3.5.4 Multimodal integration

Recent studies have explored the integration of multimodal data, including imaging, clinical, and pathologic features, into unified AI frameworks for predicting DCIS upstaging. These approaches aim to improve diagnostic accuracy and clinical decision-making compared to imaging-only models. Multimodal AI integrates diverse imaging cues such as calcifications, masses, architectural distortions, spatial features, and other subtle textural changes into a unified, quantitative abnormality score that can identify hidden invasive potential.

Yoon and coauthors used an AI-CAD system based on a ResNet-34 CNN to predict DCIS upstaging (4). They analyzed 440 DCIS cases, correlating mammographic features, BI-RADS assessments, and AI-CAD abnormality scores with final surgical pathology. Of these 26.6% of DCIS cases were upgraded to invasive carcinoma. Importantly, AI-CAD scores of ≥50% and especially ≥75% significantly predicted upgrade risk, even after adjusting for clinicopathological variables. The study concludes that AI-derived quantitative scores can serve as objective, consistent imaging biomarkers to guide pre-operative decision-making and help stratify patients who may require more aggressive management.

Park and colleagues compared four ML approaches, i.e., decision trees, bagging, and random forests, to traditional logistic regression in predicting upstaging (50). In a large cohort of 644 patients (161 upstaged, 25%), incorporating all three imaging modalities, mammography, ultrasound, and MRI, along with clinicopathologic features, the authors found that all four prediction models performed similarly with AUCs between 0.66 and 0.78, indicating no significant advantage of ML. The study identified several important predictors of upstaging. Suspicious axillary lymph nodes on ultrasound emerged as the strongest independent predictor (OR 12.16), followed by high nuclear grade; factors associated with lower upstaging risk included vacuum-assisted biopsy (OR 0.42), lesions < 2 cm on mammography (OR 0.45) or MRI (OR 0.29), and lesions that were not visible on ultrasound or MRI. Non-visible lesions on ultrasound or MRI were less likely to upgrade (P < 0.001), while non-visible lesions on X-ray showed no statistically significant association (P = 0.172). Additional imaging characteristics, such as fine linear or branching calcifications on mammography, irregular mass shape and high vascularity on ultrasound, and clustered ring enhancement or washout kinetics on MRI, were associated with higher upgrade risk on univariable analysis, although they did not remain independent predictors. Overall, the study highlights robust clinical and imaging predictors of DCIS underestimation while demonstrating that ML did not outperform conventional statistical modeling.

Similarly, Wu and colleagues developed a multimodal-DCIS-Net platform based on Inception ResNet v2 that integrated ultrasound, mammography, clinical features, and CNB pathology to better predict DCIS upstaging (1). The platform achieved an accuracy of 0.751 to 0.780 when distinguishing DCIS from upstaged DCIS (n = 141) and an AUC of 0.829–0.861, which was superior to CNB pathology accuracy of 0.688. Logistic regression outperformed random forest and achieved a higher AUC (0.861 vs. 0.829). When the platform was tested on a smaller cohort of low-grade DCIS cases (n = 33) to distinguish low-grade DCIS from upstaged low-grade DCIS, it achieved even higher performance (accuracy = 0.818–0.939; AUC = 0.769–0.987), whereas CNB accuracy remained low (0.606); in this task, random forest outperformed logistic regression in terms of accuracy (0.939 vs. 0.818, respectively) and AUC (0.987 vs. 0.769); thus, cohort size and composition influence AI model performance. Smaller datasets with more complex feature interactions may favor more flexible ML approaches such as random forests, which are able to capture nonlinearities. Across tasks, the most important predictors included US BI-RADS, MG BI-RADS, CNB nuclear grade, hormone receptor markers, US lesion type, and the deep-learning output itself, reflecting the value of integrating multimodal information. Overall, the study shows that multimodal AI can more accurately identify which DCIS lesions will be upstaged at surgery, especially for low-grade DCIS, highlighting its potential role in guiding decisions about surveillance versus surgery.

Hashiba and co-workers compared the performance of six predictive models, including logistic regression, random forest, SVM, k-nearest neighbors, gradient boosting machine, and multivariate adaptive regression spline, using multimodal data (e.g., mammography, MRI, and comprehensive clinical and pathologic features) to predict pre-surgical upstaging of DCIS in a cohort of 1,387 women with biopsy-proven pure DCIS (11). Herein, the use of a random forest model led to the highest sensitivity of 0.89 compared with 0.57-0.66 for other models and the lowest specificity of 0.19 compared with 0.38-0.50 for other models. This configuration enabled the best real-world application performance, with the highest inclusion rate in the COMET trial (87% of women) compared with 56%-69% for other models (P < 0.001 vs. COMET criteria) while preserving a similar upstaging risk (16% vs. COMET’s 12%; P = 0.15). These findings suggest that multimodal AI models can be tuned toward higher sensitivity to safely increase active surveillance inclusion although at the expense of specificity.

Collectively, multimodal integration consistently demonstrates superior or at least comparable predictive performance relative to single-modality models, with AUCs frequently exceeding 0.80 in optimized settings. Importantly, these models appear particularly valuable in refining management decisions such as eligibility for surveillance protocols rather than serving as standalone diagnostic tools. However, performance variability across cohorts and modeling approaches highlights the need for standardized feature selection, external validation, and calibration before clinical implementation.

#### 3.5.5 Differentiating microinvasion from pure DCIS

Microinvasion involves the penetration of the basement membrane by malignant cells, with a maximum invasive diameter ≤1 mm; it is usually the earliest detectable form of invasion. On the other hand, microinvasive breast carcinoma (MIBC), a broader clinical category, includes any invasive breast cancer ≤15 mm on histology. Clinically, this distinction influences tumor staging and management. Microinvasion behaves much more like DCIS whereas MIBCs, particularly those close to ≤15 mm in size, behave more like conventional early−stage invasive carcinoma that must be managed according to invasive breast cancer guidelines.

Two studies probed the use of AI in differentiating between microinvasive disease and MIBC from different angles using multimodal and deep learning approaches but both found that AI may be poised to enhance preoperative decision−making in ways that cannot be countered by traditional imaging alone (53, 54).

Vy and colleagues tested five AI models (Random Forest Classifier, Gaussian Naive Bayes, K-Nearest Neighbors, Decision Tree Classifier, and XGBoost) using multimodal data features, including mammography, ultrasonography, clinical and histopathologic features, in a total cohort of 420 patients, categorized as 189 cases of DCIS and 231 MIBC cases (53). XGBoost, a gradient boosting-based decision tree ensemble ML algorithm, achieved the best performance, reporting an accuracy of 0.84, an AUC of 0.93, and sensitivity of 0.91 for detecting MIBC, and a specificity of 0.75 for identifying DCIS, significantly outperforming the other ML methods. Thus, the model could reliably flag subtle invasive behavior that might otherwise be missed. In addition, XGBoost was tasked with identifying and ranking the top 5 features for distinguishing DCIS from MIBC; these were, ranked in order of importance, calcifications on mammograms, lymph node presence, microcalcifications on histopathology, irregular mass shape on ultrasound, and a non-parallel mass orientation on ultrasound (taller-than-wide tumors).Together, these features painted a coherent picture of how early invasion subtly alters both imaging and tissue architecture and how ensemble-based ML algorithms can integrate these cues more effectively than any single modality.

Zhu and colleagues evaluated the performance of a logistic regression model developed from ultrasound imaging data to five deep learning models (ResNet-50, ResNet-101, DenseNet-161, DenseNet-169, and Inception-v3) in the diagnosis of DCIS with microinvasion (54). The study population included 568 total patients, of which 322 patients (56.7%) were diagnosed with DCIS but no microinvasion (MIC) and 246 patients (43.3%) were diagnosed with DCIS with MIC. The Inception-v3 model performed significantly better than the other models, with the highest AUC of 0.803, an accuracy of 0.766, sensitivity of 0.767, and specificity of 0.765. However, the performance of Inception-v3 decreased on external validation, with AUC dropping from 0.803 to 0.685, highlighting generalizability challenges. Despite this drop, the study provides proof-of-concept that ultrasound-based DL may assist in automated detection of microinvasion, though robustness across institutions remains limited.

Collectively, these studies demonstrate that AI, particularly multimodal and DL models, can improve preoperative differentiation of DCIS from early invasive disease, which is critical for guiding appropriate treatment planning and surgical decision-making (53, 54).

### 3.6 AI in prediction of molecular characteristics

Integrating molecular characteristic prediction with imaging-based AI could further enhance the performance of multimodal models for DCIS management. Knowledge of receptor status, HER2 expression, and nuclear grade provides additional biologic context that may complement imaging and clinicopathologic features, potentially refining risk stratification, improving identification of lesions likely to be upstaged, and informing individualized decisions regarding active surveillance versus surgical intervention. By combining non-invasive molecular prediction with multimodal AI frameworks, future models may achieve a more comprehensive, patient-specific assessment of DCIS biology and invasive potential.

Zhu and colleagues developed and compared the feasibility of two ultrasound AI models, a deep learning radiomics (DLR) model which relied on features extracted automatically from ultrasound images using the RadImageNet pretrained network, and a clinical machine learning (CML) model, which integrated established clinically relevant variables such as patient age and ultrasound features extracted according to the 5th Edition of the Breast Imaging Reporting and Data System by experienced radiologists, for predicting molecular and histopathologic characteristics of pure DCIS (55). Specifically, they evaluated these models to predict estrogen receptor (ER), progesterone receptor (PR), human epidermal growth factor receptor 2 (HER2), and nuclear grade in a cohort of 349 patients with pure DCIS. The CML model achieved an AUC of 0.761 for ER+, 0.780 for PR+, 0.723 for HER2+, and 0.719 for low nuclear grade compared to the DLR model’s AUCs of 0.618 for ER+, 0.755 for PR+, 0.713 for HER2, and 0.633 for low nuclear grade. ER+ and nuclear grade predictions were significantly more accurate using the CML model compared to the DLR model (P = 0.01), while similar performance for PR+ (P = 0.12) and HER2+ (P = 0.12) was observed, highlighting the importance of integrating current clinical expertise in ML models.

These findings highlight the importance of integrating domain knowledge and clinical expertise into AI models, demonstrating that combining imaging features with curated clinical variables can improve predictive performance. Although ultrasound-based AI may represent a promising non-invasive approach for characterizing molecular features of DCIS, this evidence remains preliminary and is currently based on a single study. Further validation using larger, multicenter cohorts is needed before firm conclusions can be drawn regarding its role in risk stratification and individualized treatment planning.

### 3.7 AI in real-time intraoperative margin assessment

The use of CNNs for real-time intraoperative assessment of DCIS tumor margins, an emerging facet of AI integration in DCIS management that enables improved clinical and surgical decision-making and reduces positive margin rates, has also been investigated (13, 14).

Mojahed and colleagues developed a DL-based model for classifying breast tissue imaged using ultrahigh resolution optical coherence tomography (OCT); the aim was to apply this model for margin assessment in an intraoperative setting to distinguish cancerous from noncancerous regions (14). The CNN-based model was used to classify adipose, stroma, DCIS, and invasive ductal carcinoma; accuracy (94%), sensitivity (96%), and specificity (92%) were all high, indicating its applicability when used in conjunction with OCT for real-time analysis. The mean five-fold validation F1 score, used to assess the performance of image classification methods by determining similarity between 2 samples, was lower for DCIS (0.65 ± 0.15) compared with 0.89 ± 0.09 for IDC, 0.79 ± 0.17 for adipose tissue, and 0.74 ± 0.18 for stroma; however, only 3 DCIS samples were used for the study, indicating the need for larger datasets.

Li and colleagues evaluated a GoogLeNet Inception v3 architecture CNN AI model for automating tumor margin assessment when using multimodal ultrasound and photoacoustic tomography (PAT) (13). Multimodal ultrasound provided overall tissue morphology, while the integration of photoacoustic imaging enhanced visualization of adipose tissue, enabling assessment of the ≥ 2 mm clear margin requirement for DCIS, as per guidelines. The model achieved 85.5% sensitivity, 90% specificity, and an AUC of 0.93, outperforming an imaging researcher, a breast surgeon, and a board‐certified radiologist, in all imaging modality combinations. The model also processed image frames in 0.7 seconds each, with the full procedure taking approximately 10 minutes, compared to 3–5 seconds for manual reading; it also maintained significantly greater consistency (AUC 0.93 versus human inter-rater reliability scores of 0.205 to 0.565). The integration of PAT, which was performed at a 6 mm imaging depth with 13.4 mm surface adaptation, improved the detection of DCIS within 2 mm margins through enhanced visualization of lipid distribution, highlighting that the combined ultrasound and PAT system improved both sensitivity and specificity for DCIS compared to ultrasound alone.

### 3.8 AI in predicting recurrence and ipsilateral breast events after DCIS treatment

AI has also been evaluated for predicting ipsilateral breast events (IBE), including recurrence, after DCIS treatment. Lamb and coauthors developed and evaluated the use of two ML models, penalized logistic regression (LASSO) and random forest, and a traditional multivariable logistic regression model for predicting ipsilateral breast events (DCIS recurrence or IBE occurrence) after resection or treatment of DCIS (56). The models used multimodal data, including digital mammography (DM), digital mammography with tomosynthesis (DM+T), MRI, ultrasound, and a range of clinical, pathological, and treatment-related variables such as surgery type, radiation therapy, and endocrine therapy. The IBE rate was 5% with a mean follow-up of 8.0 years ±2.2 years in a cohort of 650 women with surgically treated DCIS. Traditional multivariable logistic regression, with an AUC of 0.75 performed better than both ML models, penalized logistic regression with an AUC of 0.52 and random forest with an AUC of 0.54.

Variables identified as being significant independent predictors associated with higher IBE risk, supported by multivariable analysis, included younger age, dense breasts, and <5 years of endocrine therapy. Although univariable analysis suggested higher IBE risk with breast-conserving surgery (BCS) versus mastectomy, collinearity with endocrine therapy use led the multivariable model to retain therapy duration (< 5 years vs. ≥ 5 years) rather than surgery type as the predictive factor. Importantly, this study represents a negative finding for ML in this setting: conventional multivariable logistic regression substantially outperformed both evaluated ML approaches for long-term recurrence prediction after DCIS treatment. This finding underscores the importance of benchmarking AI models against established clinical and statistical models, particularly for low-event-rate outcomes such as DCIS recurrence, where ML methods may be vulnerable to overfitting, limited outcome events, and insufficient signal.

## 4 Discussion

This review demonstrates that AI-based imaging analysis for DCIS is an expanding but heterogeneous field, with applications spanning detection, lesion classification, upstaging prediction, molecular characterization, intraoperative margin assessment, recurrence prediction, and clinical decision support. Across these domains, the most consistent finding is that DCIS remains more difficult for AI systems to detect and characterize than invasive breast cancer. Larger mammography-based studies showed that DCIS was more likely to be missed than invasive breast cancer and that AI malignancy scores for DCIS overlapped substantially with benign lesions (19, 20). Similar patterns were observed in ultrasound-based detection and MRI-based classification studies, where AI-assisted improvement or sensitivity was lower for DCIS than for invasive cancers (22, 34). Risk-prediction studies likewise showed weaker or less reliable performance for DCIS than for invasive or more advanced breast cancers (41, 42). This likely reflects the subtle and heterogeneous imaging appearance of DCIS, which may present as calcifications, non-mass enhancement, or low-conspicuity lesions rather than the more distinct mass-like findings often associated with invasive disease.

Detection studies illustrate this challenge most clearly. Some AI-CAD systems achieved high reported sensitivity for DCIS, but these findings were often based on very small DCIS subgroups, limiting generalizability (17, 18, 21, 22). Larger studies more consistently showed that DCIS was more likely to be missed than invasive breast cancer and that AI malignancy scores for DCIS overlapped substantially with benign lesions (19, 20). These findings suggest that many current AI systems are better optimized for general breast cancer detection than for DCIS-specific recognition.

Classification and risk-stratification studies provide stronger evidence for potential clinical utility, particularly when models are designed to distinguish DCIS from adjacent diagnostic entities such as IDC, ADH, fibroadenoma, fibromatosis, or microinvasive disease (15, 25–32, 53, 54). These tasks are clinically relevant because they address diagnostic uncertainty and may help guide decisions about surgery, active surveillance, or treatment intensity. However, many studies used enriched or case-control-like cohorts that may not reflect routine disease prevalence, so reported performance estimates should be interpreted cautiously and validated in unselected clinical populations.

Emerging applications such as molecular prediction, intraoperative margin assessment, and recurrence prediction remain preliminary. Ultrasound-based AI prediction of ER, PR, HER2 status, and nuclear grade may eventually complement preoperative risk stratification, but current evidence is limited to a single study (55). OCT- and photoacoustic-based intraoperative margin assessment systems showed encouraging technical performance, but DCIS-specific validation remains limited, particularly in studies with very small DCIS sample sizes or incompletely specified DCIS-specific performance (13, 14). Recurrence prediction after DCIS treatment remains especially challenging because ipsilateral breast events are uncommon and occur over long follow-up periods. In this setting, traditional multivariable logistic regression outperformed penalized logistic regression and random forest models, emphasizing that increased model complexity does not necessarily improve clinical utility (56).

### 4.1 Cross-modality evaluation of upstaging

Because prediction of occult invasion and surgical upstaging represents one of the most clinically actionable applications of AI in DCIS, modality-specific patterns were most apparent in studies focused on preoperative upstaging risk. Across these studies, mammography-based AI models generally demonstrated higher specificity but lower sensitivity, MRI-based models offered richer spatial and kinetic information but were more vulnerable to overfitting in small cohorts, ultrasound-based models showed competitive standalone performance but remained limited by operator dependence and reduced sensitivity for calcifications, and multimodal models appeared most clinically promising when imaging, clinical, and pathologic variables provided complementary rather than redundant information. More detailed information is provided in the following subsections.

#### 4.1.1 Mammography

Mammography-based models tend to demonstrate high specificity (up to ~91%) for identifying pure DCIS but relatively lower sensitivity (approximately 37–49%) for detecting invasive upstaging, depending on the selected decision threshold (12, 44). These findings may be attributed to constraints such as calcification-focused analysis. This high-specificity, low-sensitivity combination makes mammography-based models more suitable for conservative management tasks such as low-risk screening (44). Additionally, performance plateaus were reached at AUCs of 0.70 to 0.76, with transfer learning techniques matching the performance of handcrafted models, indicating diminishing returns for feature engineering within mammographic data alone (45). Clinically, mammography-based AI may therefore be most useful for high-specificity triage tasks, such as identifying low-risk patients suitable for conservative management.

#### 4.1.2 MRI

MRI-based AI models generally achieved slightly higher AUCs up to 0.796 (46) compared to mammography, with sequential models (e.g., LSTM, RRCNN) outperforming single-slice CNNs by 4–11% AUC, highlighting the value of volumetric and kinetic contrast information (46, 48). However, MRI-based AI models demonstrated a greater requirement for larger, diverse multi-institutional datasets to mitigate overfitting. For example, the MRI study by Zhu and colleagues using transfer learning via GoogleNet achieved an AUC of 0.96 on training data but only 0.53 on testing, indicating severe overfitting due to limited data (n = 131 patients) (49) while mammography models based on CNN achieved stable AUCs (approximately 0.70) with smaller datasets (n = 99 patients), suggesting less susceptibility to overfitting (45). In addition, the deep features MRI model demonstrated layer-dependent variability, with AUC ranging from 0.45–0.70 across CNN layers, further underscoring the instability of deep feature extraction in small datasets (49). Thus, while MRI offers richer biological signal through spatial and temporal enhancement patterns, it requires larger, diverse datasets to stabilize performance and mitigate overfitting.

#### 4.1.3 Ultrasound

Ultrasound-based deep learning models achieved competitive performance, particularly the ResNet-b1 model, achieving an AUC of 0.802 in predicting pre-surgical upstaging of DCIS, with a sensitivity of 0.733, specificity of 0.750, and accuracy of 0.742. The model demonstrated robustness through 3-fold cross-validation, with AUCs ranging from 0.759 to 0.817 (51).

Despite this encouraging performance, ultrasound remains limited by operator dependence and reduced sensitivity for calcifications and subtle non-mass lesions. Its predictive strength may therefore be enhanced when combined with other modalities.

#### 4.1.4 Multimodal integration

Multimodal AI models consistently demonstrated the strongest and most clinically actionable performance across upstaging tasks. Depending on the clinical objective and cohort composition, AUCs frequently exceeded 0.80 and, in selected subgroups, approached or surpassed 0.90 (1, 53). These findings suggest that integrating imaging, clinical, and pathologic features can enhance discrimination when the included variables contribute complementary predictive information rather than redundant signals.

Multimodal integration allows more flexible sensitivity tuning for active surveillance eligibility, improved prediction of low-grade DCIS upstaging, and enhanced modeling of nonlinear interactions between imaging and pathology (1, 11). However, the incremental benefit of adding clinicopathologic variables appears context-dependent rather than universal. Hou and colleagues reported that radiomic features alone achieved performance comparable to combined radiomic and clinical models (AUC 0.69 vs. 0.71), and logistic regression did not retain any of the four available clinical variables, suggesting that mammographic texture features may already encode biological correlates reflected in receptor status, nuclear grade, or age (12). In contrast, Yoon and colleagues demonstrated that high nuclear grade and radiologist-assessed BI-RADS 5 significantly improved discrimination when combined with AI-CAD scores (AUC = 0.724 vs. 0.699), indicating that certain clinicopathologic features provide independent predictive value when not redundant with imaging-derived signals (4).

Complex machine learning models did not universally outperform conventional logistic regression (50), underscoring that model sophistication must align with feature complexity, redundancy, and dataset scale. Multimodal superiority emerges not from the mere addition of variables, but from the integration of biologically complementary information that enhances risk stratification beyond what any single modality can achieve.

Multimodal models demonstrated high clinical applicability. Compared to logistic regression, random forest achieved the highest sensitivity (0.89) and real-world utility, qualifying 87% of women for active surveillance, significantly surpassing COMET criteria (42%) while maintaining comparable upstaging rates (16% vs. 12%) (11). This study illustrates how AI models can be tuned toward higher sensitivity to safely broaden surveillance inclusion without substantially increasing the risk of missed invasion.

In summary, distinct performance patterns emerge across modalities. Mammography-based AI models demonstrate high specificity and are particularly suited for conservative triage tasks, such as identifying low-risk DCIS cohorts. MRI-based AI offers greater sensitivity potential through spatial and temporal modeling of enhancement kinetics, but it is more susceptible to overfitting and often requires larger, more diverse datasets to ensure stable generalization. Ultrasound-based deep learning achieves competitive standalone performance, although it remains constrained by intrinsic modality limitations, including operator dependence and reduced sensitivity for microcalcifications. Multimodal AI appears to be the most clinically promising approach, particularly for active surveillance selection, low-grade DCIS stratification, and early invasion prediction. Traditional statistical models remain competitive in well-defined feature spaces characterized by largely linear relationships. However, AI demonstrates clear advantages when modeling nonlinear and multimodal interactions, especially in biologically complex tasks such as differentiating microinvasion from pure DCIS and refining eligibility for conservative management strategies. Collectively, the evidence suggests that the future clinical impact of AI in DCIS management will depend less on replacing established predictors and more on integrating complementary imaging and pathologic signals to enable individualized risk assessment.

### 4.2 Limitations

AI has been tested across various applications, and the results have not been very consistent, particularly for DCIS. Despite promising developments in AI for DCIS diagnosis and management, several limitations have been reported across studies, particularly concerning generalizability, dataset composition, and methodological robustness, all of which underscore key challenges in developing and implementing AI models for DCIS diagnosis and the need for careful validation and cautious clinical implementation. Our findings are broadly consistent with prior reviews of AI in breast imaging, including a review by Shamir and colleagues (7), which emphasized the promise of AI for breast cancer detection and risk stratification while highlighting persistent barriers related to dataset heterogeneity, validation, interpretability, and clinical implementation. However, the present review focuses specifically on DCIS, wherein AI development is further constrained by smaller disease-specific cohorts, subtle imaging findings, lower event rates for outcomes such as recurrence, and the need to distinguish occult invasion at diagnosis from subsequent biological progression. It is important to note here that cross-task comparisons are intended to reflect the relative maturity of the evidence base, including the number of studies, DCIS-specific sample size, validation design, and clinical applicability, rather than to imply direct superiority of one model type, modality, or clinical task over another.

A recurring limitation noted across studies was their retrospective design, which is inherently more susceptible to selection bias and confounding factors, potentially compromising the validity and generalizability of the findings. Furthermore, retrospective data often lack standardized collection protocols and may omit critical variables, thereby limiting the robustness of AI model training and evaluation in real-world clinical settings (6, 11, 30, 34, 52, 53, 56).

In some cases, the characteristics of datasets used for model training were different from those of the actual clinical population. For example, a recent study tested the performance of two DL CNN models, NYU1 and NYU2, developed at New York University for detecting breast malignancy via mammography, on an Australian dataset with and without adaptation (57). The authors found that local retraining and transfer learning with Australian datasets were required to enhance the performance and effectiveness of the original DL model. The AUC values for the two models were 0.83 and 0.89 originally, which decreased to 0.76 and 0.84 when tested on the Australian dataset. After retraining with the local dataset, the performance improved to 0.82 and 0.86, thereby demonstrating the necessity for pre-testing adaptation of AI cancer detection models with local data in a diverse clinical environment.

Hsu and colleagues conducted a similar study to externally validate a mammography-based ensemble DL model, the Challenge Ensemble Method (CEM), for automated mammography interpretation in a diverse screening population (58). The CEM model combined 11 top-performing individual AI models from the top 6 performing teams in the Digital Mammography DREAM Challenge, a collaborative, crowd-sourced initiative that aimed to improve breast cancer detection using AI. All the 11 models had been trained on the Kaiser Permanente Washington (KPW) dataset; they were tested on a new UCLA dataset for external validation to assess generalizability. Briefly, each model would indicate the likelihood of cancer by providing a confidence score between 0 and 1 that would be reweighted and aggregated to produce a combined score, thereby leveraging the strengths of each individual model. The authors compared unaltered CEM, CEM with radiologist assessment that included a binarized BI-RADS score (CEM+R), and a radiologist-only approach on a cohort of 26,817 women (37,317 exams), including 576 cancer-positive exams, of which 114 were DCIS cases (19.8%). In the UCLA external validation cohort, CEM achieved an AUC of 0.85, which was lower than its performance on KPW (AUC of 0.90) and Karolinska Institute (AUC of 0.92). CEM also underperformed compared to radiologists, with significantly lower sensitivity (0.547 vs. 0.826) and specificity (0.697 vs. 0.930); P < 0.001 in both cases. When CEM was used alongside radiologist assessments, performance improved significantly. The CEM+R assessment achieved an AUC of 0.93, with a sensitivity of 0.813 and a specificity of 0.925, which was statistically similar to radiologist performance alone (sensitivity P = 0.20; specificity P = 0.18). However, it failed to improve accuracy. Notably, the CEM+R performance also fared significantly worse in certain subgroups such as women with prior breast cancer, as the AI models confuse lumpectomy scars with cancer due to a lack of prior training on women with prior breast cancer, and women of Hispanic heritage as the models were trained predominantly on white women using the KPW and KI datasets, highlighting the importance of diverse training populations.

One of the most commonly cited limitations by most authors is the use of relatively smaller DCIS datasets, leading to challenges in AI model validation and becoming prone to performance bias (14, 15, 17, 18, 27, 46, 52, 56). Most research efforts have focused on developing AI models that include DCIS as one of several possible diagnoses, rather than as a primary target. In addition, availability of very few DCIS training cases (17, 18) may affect model development and reduce the reliability of performance estimates.

In addition to small sample size, several studies used enriched or artificially balanced class distributions that may not reflect real-world disease prevalence. For example, datasets with balanced numbers of pure DCIS and upstaged DCIS cases can improve model training and internal evaluation but may overestimate performance when applied to routine clinical populations with lower event prevalence. As a result, performance metrics from these studies should be interpreted cautiously and validated in cohorts with real-world class distributions (30, 53).

Studies have shown that cross-validation improves performance stability, but datasets with fewer than 500–700 DCIS cases often yield highly variable AUCs and unreliable model generalization (52). Hou and colleagues demonstrated the changes in mammography-based AI model performance across 50 repeated train-test splits and 200 cross-validation repeats per split when the sample size was limited (52). They compared two types of models, logistic regression and SVM, on a dataset of 700 women that comprised 586 women with pure DCIS and 114 women (16.3%) with upstaged DCIS. Logistic regression models showed more stable performance than SVMs, with less variation between training and testing results. However, they also exhibited a strong anti-diagonal tradeoff effect, i.e., an inverse relationship in performances between training and testing datasets (R² = 0.78–0.80, p < 0.05 for all logistic regression models). Thus, when training performance improved, testing performance often dropped, and vice versa. In contrast, SVM performance showed no consistent trend, with results randomly distributed around the diagonal. Models using only clinical features had the most variability, while those combining radiomics and clinical data performed better and more consistently. Importantly, the study found that cross-validation helped reduce performance bias, but it required at least 500 cases to stabilize mean AUCs, while smaller cohorts (e.g., 400 cases) had wide interquartile ranges. This study, therefore, highlights the need for larger DCIS datasets, which is difficult in most cases.

Several studies also demonstrate that conventional statistical models may perform similarly to, or in some cases better than, more complex machine-learning approaches. For example, logistic regression and other traditional multivariable models were frequently used as comparator models and sometimes showed greater stability, interpretability, or comparable discrimination relative to support vector machines, random forests, or deep learning models (1, 50, 52). This finding is important because improved model complexity does not necessarily translate into improved clinical utility, particularly in small or imbalanced DCIS datasets. Future AI studies in DCIS should, therefore, report performance against transparent conventional baselines and demonstrate incremental value beyond established clinical, imaging, and multivariable risk models.

Another frequently cited limitation is the variability introduced by heterogeneous imaging equipment and differences in user operation, which can affect diagnostic performance and AI model generalizability (34, 52, 57, 59). For example, Berg and colleagues investigated the potential for AI-supported triage of women with palpable breast lumps using portable versus standard ultrasound systems and by evaluating the scans acquired by minimally trained observers versus radiologists. The cohort included 300 women with 758 total analyzable masses, of which 56 were malignant and included 6 DCIS cases (59). Although cancer detection sensitivity remained high across systems, portable ultrasound showed significantly lower specificity (48%) as compared to standard ultrasound (79%; P < 0.001), reducing its triage effectiveness. Performance declined even further when minimally trained operators acquired the images (P = 0.04). Of the 4 malignancies misclassified as benign or probably benign, three were low-grade DCIS (appearing as circumscribed, oval, hypoechoic masses), demonstrating significantly higher misclassification rates of low−grade DCIS. While the cohort had a very small sample size of DCIS, the findings also highlight the limitations of heterogeneous imaging hardware as well as inconsistent user skill, particularly in low-resource settings, in implementing AI-based triage as part of clinical deployment.

Interestingly, studies evaluating off-label applications of commercial AI systems further underscore the importance of modality-specific model development. Lee and colleagues evaluated the performance of Lunit INSIGHT MMG (version 1.1.7), on synthetic mammography images reconstructed from digital breast tomosynthesis (60). Their study included 501 women, with 517 cancer-positive and 485 cancer-negative breasts. The AI system consistently exhibited poor performance; thus, repurposing existing tools designed for different imaging inputs may not be as efficient as modality-appropriate training and validation.

Future studies should also adhere to emerging AI-specific reporting standards, including TRIPOD-AI and CLAIM, to improve transparency in dataset construction, model development, validation, calibration, and clinical implementation. In addition, the possibility of publication bias should be considered, as studies reporting favorable AI performance may be more likely to be published than negative or inconclusive studies.

A final methodological limitation of this study is the database scope of the search strategy. Although MEDLINE Ovid, PubMed, and Web of Science were selected to capture the biomedical, radiology, oncology, and multidisciplinary literature most directly relevant to AI-based radiologic assessment of DCIS, additional AI- and engineering-focused databases, including Embase, Scopus, IEEE Xplore, and ACM Digital Library, were not searched. Therefore, some technical AI studies relevant to DCIS imaging may not have been captured.

Nevertheless, the findings from the included studies highlight that while AI holds substantial potential for DCIS management, its clinical utility is constrained by dataset limitations, population diversity, standardization of imaging and clinical inputs, and the need for rigorous external validation. Addressing these limitations will be critical for translating AI advances into safe, reliable, and equitable clinical practice.

## 5 Conclusions

The current evidence highlights both the potential and the critical gaps in AI-based DCIS detection and management. AI has demonstrated potential to enhance multiple aspects of care, including differential diagnosis, intraoperative margin assessment, grade categorization, presurgical upstaging prediction, prediction of ipsilateral breast events after resection, and molecular characterization using imaging, particularly ultrasound. These applications could significantly improve patient stratification and guide personalized management strategies.

However, substantial challenges remain. Across applications, AI performance for DCIS appears less robust than for invasive breast cancer, emphasizing the need for DCIS-specific datasets, validation, and cautious clinical translation. Most studies are retrospective, include relatively small DCIS cohorts, and often lack robust external or cross-validation, limiting generalizability. Current AI models frequently rely on heterogeneous datasets or off-label applications, highlighting the need for DCIS-specific algorithm development and modality-appropriate validation.

Future research should prioritize larger, more diverse, multi-institutional datasets, explicitly DCIS-focused model construction, and improved interpretability of complex AI systems. By addressing these gaps, AI has the potential to transition from a research tool to a clinically reliable adjunct, enabling individualized risk assessment and ultimately improving patient outcomes in DCIS management.

## Data Availability

The original contributions presented in the study are included in the article/**Supplementary Material**. Further inquiries can be directed to the corresponding author.
