# Supplementary material for: Artificial intelligence in the radiologic assessment of ductal carcinoma in situ: a systematic review
Source: Front Oncol. 2026 Jul 14;16:1870768. doi: 10.3389/fonc.2026.1870768 (PMC13407775; doi:10.3389/fonc.2026.1870768)
Supplement: Supplementary file 1 [file Table1.docx]

**Supplementary Table 1: Risk of bias analysis using QUADAS-2**

| **Study (Author, Year)** | **Patient Selection (RoB)** | **Index Test (RoB)** | **Reference Standard (RoB)** | **Flow & Timing (RoB)** | **Applicability: Patient Selection** | **Applicability: Index Test** | **Applicability: Reference Standard** |
| --- | --- | --- | --- | --- | --- | --- | --- |
| Alaeikhanehshir et al., 2024 [5] | High | Low | Low | Low | Low | Low | Low |
| Barros et al., 2023 [35] | High | Low | Low | Low | High | Low | Low |
| Berg et al., 2023 [59] | Low | Low | Low | Low | Low | Low | Low |
| Condon et al., 2024 [57] | Low | Low | Low | Low | Low | Low | Low |
| Damiani et al., 2023 [41] | Low | Low | Low | Low | Low | Low | Low |
| Do et al., 2022 [46] | High | Low | Low | Low | Low | Low | Low |
| Ha et al., 2019 [30] | High | High | Low | Low | High | Low | Low |
| Hashiba et al., 2023 [11] | High | High | Low | Low | Low | Low | Low |
| Hou et al., 2022 [12] | High | Low | Low | Low | Low | Low | Low |
| Hou et al., 2024 [52] | High | High | Low | Low | Low | Low | Low |
| Hsu et al., 2022 [58] | Low | Low | Low | Low | Low | Low | Low |
| Jayender et al., 2013 [25] | High | High | Low | Low | High | Low | Low |
| Jiang et al., 2024 [32] | High | Low | Low | Low | High | Low | Low |
| Lamb et al., 2022 [56] | High | Low | Low | High | High | Low | Low |
| Lee H-J. et al., 2022 [47] | High | Low | Low | Low | Low | Low | Low |
| Lee K. E. et al., 2025 [60] | Low | Low | Low | Low | High | High | Low |
| Lee S. E. et al., 2024 [6] | High | Low | Low | High | High | Low | Low |
| Lee S.E., 2022 [20] | High | High | Low | High | High | Low | Low |
| Li et al., 2018 [13] | High | Low | Low | Low | High | High | Low |
| Liu C. et al., 2024 [36] | High | High | Low | Low | High | Low | Low |
| Liu Y. et al., 2024 [34] | High | Low | Low | Low | High | Low | Low |
| Ma et al., 2024 [22] | Low | Unclear | Low | Low | Low | Low | Low |
| Manley et al., 2021 [43] | High | Low | Low | High | High | Low | Low |
| Mayfield et al., 2024 [48] | High | Low | Low | Low | Low | Low | Low |
| Mojahed et al., 2020 [14] | High | Low | Low | Low | High | Low | Low |
| Mutasa, Chang, Nemer, et al., 2020 [15] | High | Low | Low | Low | High | Low | Low |
| Mutasa, Chang, Van Sant, et al., 2020 [44] | High | High | Low | Low | Low | Low | Low |
| Nassif et al., 2012 [26] | High | High | Low | Low | High | Low | Low |
| Park et al., 2022 [50] | High | High | Low | Low | Low | Low | Low |
| Qian et al., 2021 [51] | High | High | Low | Low | Low | Low | Low |
| Raafat et al., 2022 [17] | High | High | Low | Low | High | Low | Low |
| Santeramo et al., 2024 [42] | Low | Low | Low | Low | Low | Low | Low |
| Shi et al., 2018 [45] | High | High | Low | Low | Low | Low | Low |
| Vy et al., 2022 [53] | High | Low | Low | Low | Low | Low | Low |
| Waugh et al., 2024 [18] | Low | Low | Low | Low | Low | Low | Low |
| Weigel et al., 2023 [19] | Low | Low | Low | Low | Low | Low | Low |
| Wu Y et al., 2025 [33] | High | Low | Low | Low | High | Low | Low |
| Wu H. et al., 2024 [1] | High | Low | Low | Low | Low | Low | Low |
| Xiao et al., 2019 [21] | High | High | Low | Low | High | Low | Low |
| Yin H. et al., 2021 [27] | High | Low | Low | Low | High | Low | Low |
| Yin J. et al., 2023 [31] | High | Low | Low | Low | High | Low | Low |
| Yoon et al., 2024 [4] | High | Low | Low | Low | Low | Low | Low |
| Zhang et al., 2024 [28] | High | Low | Low | Low | High | Low | Low |
| Zhu Z. et al., 2019 [49] | High | High | Low | Low | Low | Low | Low |
| Zhu M. et al., 2022 [54] | High | High | Low | Low | Low | Low | Low |
| Zhu M. et al., 2024 [55] | High | Low | Low | Low | High | Low | Low |
